# β-Amyloid in blood neuronal-derived extracellular vesicles is elevated in cognitively normal adults at risk of Alzheimer’s disease and predicts cerebral amyloidosis

**DOI:** 10.1186/s13195-022-01010-x

**Published:** 2022-05-12

**Authors:** Tao-Ran Li, Yun-Xia Yao, Xue-Yan Jiang, Qiu-Yue Dong, Xian-Feng Yu, Ting Wang, Yan-Ning Cai, Ying Han

**Affiliations:** 1 Department of Neurology, Xuanwu Hospital of Capital Medical University, Beijing, 100053 China; 2 Department of Neurobiology, Xuanwu Hospital of Capital Medical University, Beijing, 100053 China; 3 School of Biomedical Engineering, Hainan University, Haikou, 570228 China; 4 Key Laboratory of Specialty Fiber Optics and Optical Access Networks, Joint International Research Laboratory of Specialty Fiber Optics and Advanced Communication, School of Information and Communication Engineering, Shanghai University, Shanghai, 200444 China; 5 Center of Alzheimer’s Disease, Beijing Institute for Brain Disorders, Beijing, 100053 China; 6 National Clinical Research Center for Geriatric Diseases, Beijing, 100053 China

**Keywords:** Alzheimer’s disease, Preclinical AD, Extracellular vesicle, nEVs, Blood biomarker, Aβ

## Abstract

**Background:**

Blood biomarkers that can be used for preclinical Alzheimer’s disease (AD) diagnosis would enable trial enrollment at a time when the disease is potentially reversible. Here, we investigated plasma neuronal-derived extracellular vesicle (nEV) cargo in patients along the Alzheimer’s continuum, focusing on cognitively normal controls (NCs) with high brain β-amyloid (Aβ) loads (Aβ+).

**Methods:**

The study was based on the Sino Longitudinal Study on Cognitive Decline project. We enrolled 246 participants, including 156 NCs, 45 amnestic mild cognitive impairment (aMCI) patients, and 45 AD dementia (ADD) patients. Brain Aβ loads were determined using positron emission tomography. NCs were classified into 84 Aβ− NCs and 72 Aβ+ NCs. Baseline plasma nEVs were isolated by immunoprecipitation with an anti-CD171 antibody. After verification, their cargos, including Aβ, tau phosphorylated at threonine 181, and neurofilament light, were quantified using a single-molecule array. Concentrations of these cargos were compared among the groups, and their receiver operating characteristic (ROC) curves were constructed. A subset of participants underwent follow-up cognitive assessment and magnetic resonance imaging. The relationships of nEV cargo levels with amyloid deposition, longitudinal changes in cognition, and brain regional volume were explored using correlation analysis. Additionally, 458 subjects in the project had previously undergone plasma Aβ quantification.

**Results:**

Only nEV Aβ was included in the subsequent analysis. We focused on Aβ_42_ in the current study. After normalization of nEVs, the levels of Aβ_42_ were found to increase gradually across the cognitive continuum, with the lowest in the Aβ− NC group, an increase in the Aβ+ NC group, a further increase in the aMCI group, and the highest in the ADD group, contributing to their diagnoses (Aβ− NCs vs. Aβ+ NCs, area under the ROC curve values of 0.663; vs. aMCI, 0.857; vs. ADD, 0.957). Furthermore, nEV Aβ_42_ was significantly correlated with amyloid deposition, as well as longitudinal changes in cognition and entorhinal volume. There were no differences in plasma Aβ levels among NCs, aMCI, and ADD individuals.

**Conclusions:**

Our findings suggest the potential use of plasma nEV Aβ_42_ levels in diagnosing AD-induced cognitive impairment and Aβ+ NCs. This biomarker reflects cortical amyloid deposition and predicts cognitive decline and entorhinal atrophy.

**Supplementary Information:**

The online version contains supplementary material available at 10.1186/s13195-022-01010-x.

## Introduction

Currently, Alzheimer’s disease (AD) remains the only leading cause of death without an available disease-modifying therapy. It is characterized by the co-existence of aberrantly accumulated amyloid-β (Aβ) and hyperphosphorylated tau [1]. According to the latest diagnostic frameworks [2], individuals exhibiting evidence of brain Aβ deposition have already entered the Alzheimer’s continuum, indicating a high risk of AD. Due to its incurable and irreversible nature, it is of great importance to recognize AD patients at the ultra-early stage and carry out specific interventions [3, 4]. Although cerebrospinal fluid (CSF) detection and positron emission tomography (PET) imaging have made great progress [2], there is still an urgent and unmet need for convenient and cost-effective early diagnostic biomarkers.

The discovery of extracellular vesicles (EVs) has greatly improved our understanding of cell-to-cell communication. EVs facilitate the accumulation and spread of AD-associated toxic cargo, while enhancing intercellular communication [5]. During this process, some EVs are likely to cross the blood–brain barrier into the peripheral blood [6], making them potential carriers of biomarkers. Additionally, EVs can reflect the state of their source cells [7], and the successful isolation of blood–brain-derived EVs further enhances this possibility [8, 9]. In our previous reviews [5, 10], we have summarized the role of EVs as AD biomarkers. Briefly, brain-derived EVs, such as neuronal-derived EVs (nEVs), which are present in the blood, carry many different types of cargo, including Aβ [9], phosphorylated tau [9], synapse-related proteins [11], and other molecules [8, 12], and can be used to diagnose AD. Furthermore, a recent study suggested that Aβ_42_, tau phosphorylated at threonine 181 (p-tau181), and t-tau levels in nEVs are closely related to those in the CSF [13]. Similar results have been obtained in AD mouse models, where biomarkers in circulating nEVs were strongly and positively correlated with their levels in the brain [14]. Additional file 1: Table S1 lists previous studies on nEV Aβ and tau as biomarkers of AD. However, several unresolved issues remain. First, the findings regarding the preclinical stage of AD are conflicting [9, 12]: it remains unclear whether the cargos (particularly Aβ) in nEVs have really changed at this early stage. Second, no study has explored the relationship between nEVs and neuroimaging (amyloid-PET, structural magnetic resonance imaging [sMRI], etc.) or cognition. Exploring these questions will facilitate early diagnosis of AD and prediction of outcome events, which are particularly meaningful for clinical research.

The goals of this study were as follows: (1) to explore the dynamic changes in AD-related proteins, such as Aβ, carried in nEVs, in the Alzheimer’s continuum, with a focus on cognitively normal controls (NCs) with high brain Aβ loads (Aβ+) and (2) to evaluate the relationships between nEV cargo and brain Aβ deposition (reflected by amyloid-PET), brain regional volume (reflected by sMRI), and cognition. In addition, we quantified the plasma Aβ levels of some participants to make horizontal comparisons.

## Participants and methods

### Participants

Participants were enrolled in the Sino Longitudinal Study on Cognitive Decline (SILCODE, ClinicalTrials.gov identifier: NCT03370744) from December 2015 to May 2021. The SILCODE project is a registered ongoing multicenter AD study in the Han population of mainland China [15]. Each subject provided detailed baseline clinical information, including demographic data, apolipoprotein E (APOE) status, and results of a battery of neuropsychological tests, including the auditory verbal learning test (AVLT), animal fluency test (AFT), 30-item Boston naming test (BNT), shape trails test (STT)—parts A and B, Mini-Mental State Examination (MMSE), Montreal Cognitive Assessment-Basic (MoCA-B), and Clinical Dementia Rating scale (CDR). The details can be obtained from the protocol [15] and from our previous studies [16, 17].

NCs were diagnosed based on the exclusion of mild cognitive impairment (MCI) [18, 19] and dementia [20], requiring a CDR score of 0, no obvious emotional problems, and normal education-adjusted scores in the MMSE and memory subdomain. A subset of NCs had a subjective cognitive decline. They were analyzed together with cognitively healthy participants, in accordance with the research framework of the National Institute on Aging–Alzheimer’s Association [2]. MCI diagnosis was based on neuropsychological criteria [19]. The amnestic MCI (aMCI) subtype required an impaired memory subdomain. The entry criterion for AD dementia (ADD) referred to the proposed criteria for probable AD-induced dementia [20]. In our study, NCs were further classified as Aβ+ according to a priori principles and our previous studies that utilized an established cortical [^18^F]florbetapir (AV45) standardized uptake value ratio (SUVR) cutoff > 1.18 [16, 17, 21, 22]. The remaining NCs were classified as Aβ−. In comparison, amyloid-PET is not necessary to diagnose aMCI or ADD, but we stipulated that, in those subjects who had undergone PET examination, Aβ deposition had to be obvious. Ultimately, 84 Aβ− NCs, 72 Aβ+ NCs, 45 patients with aMCI, and 45 patients with ADD were enrolled. Among them, 51.6% were included in 2018; we did not include new subjects due to the impact of corona virus disease 2019 in 2020.

### Brain imaging

Amyloid-PET and sMRI data were obtained using an integrated simultaneous 3.0-T time-of-flight PET/MRI system (SIGNA, GE Healthcare, Chicago, IL, USA). All NCs and 25.6% of the cognitive impairment patients had amyloid-PET data, with an average interval between PET scans and plasma collection of 40.4 ± 42.1 days (mean ± standard deviation [SD]). We acquired the global and regional AV45 SUVR of each participant using the same methods as in our previous studies [16, 17, 21, 23]. Most of the subjects (78.0%) had baseline sMRI data, with an average interval between sMRI scans and plasma collection of 18.9 ± 34.8 days (mean ± SD). The sMRI data were processed using the CAT12 toolbox (http://dbm.neuro.uni-jena.de/cat/), within the SPM12 software (www.fil.ion.ac.uk/spm) on the MATLAB R2016b platform (MathWorks, Natick, MA, USA). Here, we mainly focused on the hippocampus, entorhinal cortex, posterior cingulate cortex (PCC), and precuneus (because these are typical regions with early AD-related pathological protein deposition and neurodegeneration [1, 24]), as well as the total intracranial volume (TIV) and gray matter (GM) volume.

Details regarding the imaging acquisition protocol and processing steps are provided in Additional file 1: Supplementary material.

### Isolation of nEVs from plasma

The participants provided blood samples at the time of clinical evaluation. Blood samples were collected in EDTA polypropylene tubes in the morning after an overnight fast. After centrifugation (2500 rpm, 15 min, 4 °C, twice), the supernatant plasma was aliquoted and stored at − 80 °C in the clinical sample center of Xuanwu Hospital. Each sample had undergone 1–2 freeze–thaw cycles before use. We precipitated EVs using Exoquick® and further enriched nEVs using an anti-CD171 antibody. The isolation process has been described previously [9, 12], with some modifications. The details are provided in Additional file 1: Supplementary material.

### nEV characterization

We performed transmission electron microscopy (TEM) to characterize the morphology of single nEVs, nanoparticle tracking analysis (NTA) to calculate EV concentration and average diameter, and western blotting to verify the nature (CD63, TSG101), purity (Albumin, GM130), and neuronal origin (Tubb3, SNAP25) of EVs, following the guidance of MISEV2018 [25]. The details are described in Additional file 1: Supplementary material.

### Plasma Aβ quantification

Previously, 458 subjects in the SILCODE project underwent plasma Aβ quantification; their clinical characteristics are displayed in Additional file 1: Table S3, and some results have been disclosed [26]. Among these, 81 Aβ− NCs, 45 Aβ+ NCs, 34 subjects with aMCI, and 20 subjects with ADD were included in the current nEV study. The measurements were based on an electrochemiluminescence method (K15199E; Meso Scale Discovery [MSD], Rockville, MD, USA). All assays were conducted in duplicate, and the quality control is shown in Additional file 1: Supplementary material. These data were analyzed to provide a horizontal comparison.

### nEV protein quantification

Our pre-experiment results suggested that the electrochemiluminescence method was not sufficiently sensitive to detect Aβ and t-tau in nEVs (K15199E and K15121D, respectively; MSD; data not shown). Therefore, we used two single-molecule array kits (Simoa; Quanterix, Billerica, MA, USA): Neurology 4-Plex E and pTau-181 V2, to measure nEV proteins. Notably, compared to previous single-factor or tri-factor kits, the former was newly developed for highly specific and sensitive measurement of the concentrations of full-length Aβ_1–42_ and Aβ_1–40_ [27]. All assays were conducted in duplicate, and the quality control is described in Additional file 1: Supplementary material and shown in Additional file 1: Table S2. Unexpectedly, based on quality control, p-tau181 and neurofilament light (NFL) results were both excluded, and only Aβ_40_ and Aβ_42_ results were included in the subsequent analysis. We did not analyze glial fibrillary acidic protein, as this is an astrocytic marker.

### Statistical analysis

In Table 1 and Additional file 1: Table S3, the demographic, neuropsychological, and imaging data and plasma Aβ concentration are summarized as numbers (%) or as means ± SDs for categorical and continuous variables, respectively. Chi-square tests were used to compare the categorical variables. Independent two-sample *t*-tests were used for STT-A/B, AFT, and BNT scales. Kruskal–Wallis *H* tests followed by multiple post hoc comparisons were used for other continuous variables.

**Table 1:** Baseline characteristics of enrolled subjects

Groups	Aβ− NCs	Aβ+ NCs	aMCI	ADD
***N***	84	72	45	45
**Age (years)**	65.3 ± 5.5	67.2 ± 6.6^#^	69.6 ± 6.8**	73.9 ± 8.8***
**Male**	28 (33.3%)	24 (33.3%)^#^	21 (46.7%)^#^	15 (33.3%)^#^
**Education**	12.3 ± 3.3	13.4 ± 3.2^#^	11.0 ± 4.1^#^	10.8 ± 4.5^#^
**MMSE (out of 30)**	28.6 ± 1.7	28.7 ± 1.7^#^	24.1 ± 3.4***	17.3 ± 5.3***
**MoCA-Basic (out of 30)**	25.7 ± 2.3	26.6 ± 2.4^#^	20.1 ± 2.9***	11.6 ± 4.4***
**AVLT-N5**	7.7 ± 1.9	7.7 ± 2.3^#^	2.0 ± 1.6***	0.8 ± 1.2***
**AVLT-N7**	22.4 ± 1.6	22.6 ± 1.4^#^	16.9 ± 2.1***	15.0 ± 2.9***
**STT-A**	56.7 ± 16.4	55.9 ± 17.1^#^	NA	NA
**STT-B**	134.3 ± 38.4	131.3 ± 40.7^#^	NA	NA
**AFT**	18.4 ± 4.7	19.6 ± 5.0^#^	NA	NA
**BNT**	24.9 ± 3.3	25.8 ± 2.9^#^	NA	NA
***APOE*** **ε4 carries**	23 (27.4%)	32 (44.4%)^#^	24 (53.3%)**	31 (68.9%)***
**AV45 SUVR**	1.096 ± 0.059	1.241 ± 0.060***	1.390 ± 0.085 (11Ava)***	1.419 ± 0.084 (12Ava)***
**sMRI**	75Ava (89.3%)	62Ava (86.1%)	27Ava (60.0%)	28Ava (62.2%)
**Hp/TIV ratio**	6.596 ± 1.944	6.304 ± 1.892^#^	5.534 ± 2.444*	4.066 ± 3.012***
**Ent/TIV ratio**	4.226 ± 1.251	3.933 ± 1.206^#^	3.479 ± 1.217*	2.317 ± 0.834***
**PCC/TIV ratio**	3.280 ± 1.840	3.456 ± 1.797^#^	3.651 ± 1.730^#^	4.320 ± 1.098^#^
**Pre/TIV ratio**	8.941 ± 4.337	9.340 ± 4.287^#^	9.557 ± 4.316^#^	11.337 ± 2.722^#^

Data were summarized as numbers (%) or as means ± standard deviations for categorical and continuous variables, respectively. Some patients with aMCI and ADD were enrolled before the end of 2016; they did not undergo the STT, AFT, and BNT scales, and some patients could not cooperate with and/or understand these tests. Thus, the results of these two groups are not listed. Indicators of sMRI were presented as the ratio of the regional volume to the TIV, multiplied by a factor of 1000. Statistical analyses were conducted using the chi-square test for categorical variables and the Kruskal–Wallis *H* test for continuous variables (independent two-sample *t*-test for STT-A/B, AFT, and BNT), followed by multiple post hoc comparisons (adjusted *p* value). Compared with the Aβ− NCs: *p < 0.05; **p < 0.01; ***p < 0.001; ^#^> 0.05

*Abbreviations*: *Aβ* β-amyloid, *NCs* cognitively normal controls, *aMCI* amnestic mild cognitive impairment, *ADD* Alzheimer’s disease dementia, *MMSE* Mini-Mental State Examination, *MoCA-B* Montreal Cognitive Assessment-Basic Version, *AVLT* auditory verbal learning test, *N5* AVLT-delayed memory, *N7* AVLT-recognition, *STT* shape trails test, *AFT* animal fluency test, *BNT* Boston naming test, *APOE* apolipoprotein E, *AV45* [^18^F]florbetapir, *SUVR* standardized uptake value ratio, *sMRI* structural magnetic resonance imaging, *TIV* total intracranial volume, *HP* hippocampus, *Ent* entorhinal cortex, *PCC* posterior cingulate cortex, *Pre* precuneus, *Ava* available, *NA* not available

For group comparisons of NTA results (particle concentration, diameter, etc.) and Aβ concentrations, we performed Kruskal–Wallis *H* tests followed by multiple post hoc comparisons. Differences in nEV Aβ levels between the two NC groups were further verified after correcting for confounding factors, including age, sex, and *APOE* ε4 status. The area under the receiver operating characteristic (ROC) curve (AUC) values, with 95% confidence intervals (CIs), were used to evaluate the ability of the indicators to distinguish Aβ− NCs or NCs from other groups.

To explore whether correlations existed between the levels of Aβ_40_ and Aβ_42_, Aβ and cognition, and Aβ and imaging markers, including global brain Aβ deposition and brain regional volumes, Spearman correlation coefficients were calculated. Linear regression models were used to evaluate the above associations further with adjustment for confounding factors (see legends for details). In addition, we used partial correlation analyses to evaluate the relationships between nEV Aβ levels and regional Aβ deposition after correcting for age, sex, and *APOE* ε4 status. Here, cognition was represented by the MMSE and MoCA-B scales, and the regional volume was expressed as the ratio to the TIV. However, in the regression models, the TIV was used as a covariate. Notably, we also evaluated the associations between baseline Aβ concentrations and longitudinal changes in cognition or regional volumes in a subset of participants. The processing and analysis of longitudinal data were based on previous studies [28, 29]. Briefly, the longitudinal changes were represented as the magnitude of changes in scales or volumes, the latter was annualized, and the associations were assessed using Spearman correlation and linear regression analyses. Considering the incompleteness of the data, we compared demographic data between the participants with and without baseline sMRI, longitudinal sMRI, or longitudinal cognitive evaluations (Additional file 1: Table S4) and found no differences between these groups.

The significance threshold was set at *p* < 0.05, and the above analyses were performed using SPSS v24 (SPSS Inc., Chicago, IL, USA) or R software, version 4.0.1.

## Results

### Subject characteristics

Two hundred forty-six participants were included in this study: 84 Aβ− NCs, 72 Aβ+ NCs, 45 aMCI, and 45 ADD. Table 1 describes the baseline demographic and clinical characteristics of this cohort, categorized by diagnosis. As expected, there were no differences between Aβ− NCs and Aβ+ NCs, except for AV45 SUVR. The ADD and aMCI patients both had a higher mean age than the Aβ− NCs, but there were no differences among the groups with regard to sex or education. The scores of cognitive scales, including the MMSE, MoCA-B, and AVLT, gradually decreased across the cognitive continuum, with the highest scores observed in the NC groups, decreased scores in the aMCI group, and the lowest scores in the ADD group. In addition, patients with cognitive impairment were more likely to carry the *APOE* ε4 allele. Amyloid-PET was available for all NCs and for some patients with aMCI (24.4%) or ADD (26.7%). The aMCI and ADD groups both showed higher AV45 SUVR than did Aβ− NCs. Baseline sMRI was available for 89.3% of the Aβ− NCs, 86.1% of the Aβ+ NCs, 60.0% of the subjects with aMCI, and 62.2% of the subjects with ADD. Compared to NCs, patients with cognitive impairment demonstrated more atrophy in the hippocampus and entorhinal cortex. In contrast, there was no obvious atrophy in the PCC and precuneus, and the apparent upward trends were caused by a significant decrease in the TIV.

### nEV Aβ concentrations and ROC analysis

We followed MISEV2018 requirements to verify the extracted nEVs [25]. More specifically, nEVs were first analyzed for morphology using TEM (Additional file 1: Fig. S1), which revealed a population of particles with saucer-like morphology and a clear membrane structure of approximately 150-nm diameter. Second, as shown in Additional file 1: Figs. S2 and S3, the sizes and concentrations of nEVs were directly determined by NTA. The average diameters of nEVs were the same among the four groups. However, the concentrations of these particles in patients with aMCI or ADD were lower than those in Aβ− NCs or Aβ+ NCs. Third, the nature of EVs and purity of plasma nEVs were validated by western blotting, using two positive markers (CD63 and TSG101) and two negative markers (albumin and GM130). Additionally, TUBB3 and SNAP25, two classic neuronal markers, were clearly observed in nEVs, suggesting a true neuronal origin for these particles (Additional file 1: Fig. S4).

**Fig. 1:**
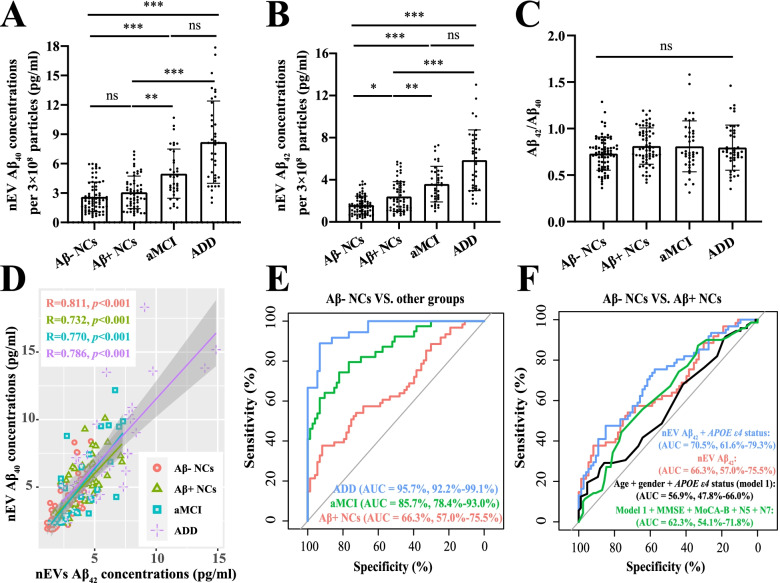
nEV Aβ concentrations in different diagnostic groups and ROC curves. Analyses performed after correcting the particle numbers to 3 × 10^8^ for nEV Aβ_40_ (**A**) and nEV Aβ_42_ (**B**). **C** The ratio of Aβ_42_ to Aβ_40_ among the different diagnostic groups. Statistical analysis was conducted using the Kruskal–Wallis *H* test, followed by multiple post hoc comparisons (adjusted *p* value). Comparisons among the groups: *p < 0.05; **p < 0.01; ***p < 0.001; ns, > 0.05. Furthermore, correlation analysis was performed between Aβ_42_ and Aβ_40_ (**D**), and the correlation coefficients and *p* values are listed. **E** ROC curves used to distinguish Aβ− NCs from Aβ+ NCs (red), aMCI (green), and ADD (blue) using nEV Aβ_42_ levels. **F** We attempted to use different combinations to distinguish Aβ− NCs from Aβ+ NCs. AUC and its corresponding 95% CIs and different diagnostic combinations are listed. All outliers were excluded; outliers were defined as less than Q1 − 2.5 × IQR or greater than Q3 + 2.5 × IQR. Aβ, β-amyloid; NCs, cognitively normal controls; aMCI, amnestic mild cognitive impairment; ADD, Alzheimer’s disease dementia; nEV, neuronal-derived extracellular vesicle; AUC, area under the curve; ROC, receiver operating characteristic; CI, confidence interval; IQR, inter-quartile range; Q1, lower quartile; Q3, upper quartile

Since the efficiency of nEV isolation could vary across samples and might mask the differences in the amount of Aβ measured, and considering that most previous studies had made comparisons only after correction [9, 11–13], we normalized measurements of Aβ in each sample relative to that of the established particle concentrations. Figure 1A, B shows the Aβ_40_ and Aβ_42_ results, respectively, after correcting for the particle number to 3 × 10^8^. More specifically, the nEV Aβ_42_ levels gradually increased across the cognitive continuum, with the lowest in the Aβ− NC group (1.592 ± 0.852 pg/ml), an increase in the Aβ+ NC group (2.406 ± 1.417 pg/ml; vs. Aβ− NC, *p* < 0.05), a further increase in the aMCI group (3.593 ± 1.699 pg/ml; vs. Aβ+ NC, *p* < 0.01), and reaching the highest in the ADD group (5.853 ± 2.880 pg/ml; vs. aMCI, borderline statistically significant differences with *p* = 0.086). Importantly, the differences between the NC groups remained after adjusting for age, sex, and *APOE* genotype (*p* = 0.002; Additional file 1: Table S5). Aβ_40_ showed similar results, with the exception that there were no significant differences between the NC groups. All outliers were deleted in Fig. 1, and retaining these values had no significant effects on the results (Additional file 1: Fig. S5). Furthermore, we found that Aβ_40_ and Aβ_42_ concentrations were significantly and positively correlated (Fig. 1D), and there was no difference in their ratios among the groups (Fig. 1C). Considering their strong correlation and the importance of Aβ_42_, subsequent analyses mainly focused on Aβ_42_.

As shown in Fig. 1E, nEV Aβ_42_ levels showed excellent ability to distinguish aMCI or ADD individuals from Aβ− NCs, with AUCs of 0.857 and 0.957, respectively, but not for identifying Aβ+ NCs (AUCs of 0.663), irrespective of whether outliers were included or excluded (Additional file 1: Fig. S5C). Figure 1F shows the results of the different combinations used to identify the two NC groups. Specifically, the addition of the *APOE* genotype slightly increased the AUC from 0.663 to 0.705 (DeLong test: *p* = 0.09), whereas adding demographic characteristics, with or without clinical scales, performed poorly (AUCs of 0.569 and 0.623). The use of sMRI indicators was not helpful for identification (AUCs < 0.60). Compared to the dementia stage, the aMCI stage also has therapeutic potential. As shown in Table 2, the ability of nEV Aβ_42_ levels to discriminate aMCI from NCs was not markedly weakened with the inclusion of Aβ+ NCs (AUC 0.791) and was higher than that of the “demographic model” (AUC 0.657). Similar results were obtained for distinguishing ADD individuals from NCs.

**Table 2:** ROC curves

Categorical variables	AUC (Aβ− NCs vs. aMCI)	AUC (NCs vs. aMCI)	AUC (NCs vs. ADD)
**nEV Aβ**_**42**_	85.67% (78.36–92.98%)	79.12% (71.33–86.92%)	91.48% (86.76–96.2%)
**nEV Aβ**_**42**_ **+** ***APOE*** **ε4 status**	87.39% (80.39–94.39%)	79.97% (72.08–87.85%)	90.4% (84.74–96.06%)
**Age + sex +** ***APOE*** **ε4 status (model 1)**	70.72% (59.66–81.79%)	65.74% (55.12–76.35%)	76.6% (64.64–88.55%)
**Model 1 + MMSE + MoCA-B + N5 + N7**	96.77% (94.08–99.47%)	97.25% (95.17–99.33%)	99.78% (99.45–100%)

ROC curves were used to distinguish Aβ− NCs from aMCI individuals, NCs from aMCI individuals, and NCs from ADD individuals. AUC and its corresponding 95% CIs are listed

*Abbreviations*: *Aβ* β-amyloid, *NCs* cognitively normal controls, *aMCI* amnestic mild cognitive impairment, *ADD* Alzheimer’s disease dementia, *MMSE* Mini-Mental State Examination, *MoCA-B* Montreal Cognitive Assessment-Basic Version, *N5* auditory verbal learning test-delayed memory, *N7* auditory verbal learning test-recognition, *APOE* apolipoprotein E, *nEV* neuronal-derived extracellular vesicle, *AUC* area under the curve, *ROC* receiver operating characteristic, *CI* confidence interval

### Plasma Aβ

**Fig. 2:**
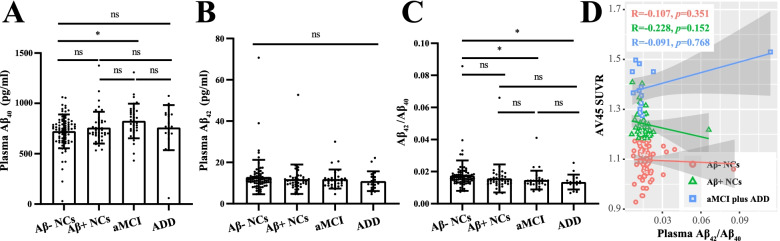
Plasma Aβ concentrations in different diagnostic groups. In the current nEV study, 180 subjects (81 Aβ− NCs, 45 Aβ+ NCs, 34 aMCI, and 20 ADD individuals) had previously undergone plasma Aβ_40_ (**A**) and Aβ_42_ (**B**) quantification. **C** The ratio of Aβ_42_ to Aβ_40_ among the different diagnostic groups. Statistical analysis was conducted using the Kruskal–Wallis *H* test, followed by multiple post hoc comparisons (adjusted *p* value). Comparisons among groups: *p < 0.05; ns, > 0.05. Furthermore, a correlation analysis was performed between the Aβ_42_ to Aβ_40_ ratio and AV45 SUVR among the different groups (**D**). The correlation coefficients and *p* values are listed. Aβ, β-amyloid; NCs, cognitively normal controls; aMCI, amnestic mild cognitive impairment; ADD, Alzheimer’s disease dementia; nEV, neuronal-derived extracellular vesicle; AV45, [^18^F]florbetapir; SUVR, standardized uptake value ratio

In contrast, plasma Aβ_42_ and Aβ_40_ levels and their ratios exhibited no differences among clinically diagnosed NCs, aMCI, and ADD individuals (Additional file 1: Fig. S6). A total of 180 patients were included in the current nEV study. The Aβ− NCs had higher Aβ_42_/Aβ_40_ ratios than those in patients with aMCI or ADD (*p* < 0.05); however, huge overlaps were observed among these groups (details are shown in Fig. 2). Importantly, correlation analyses indicated that plasma Aβ levels were not related to brain Aβ deposition, as represented by AV45 SUVR (Fig. 2D).

### Relationships with amyloid-PET

**Fig. 3:**
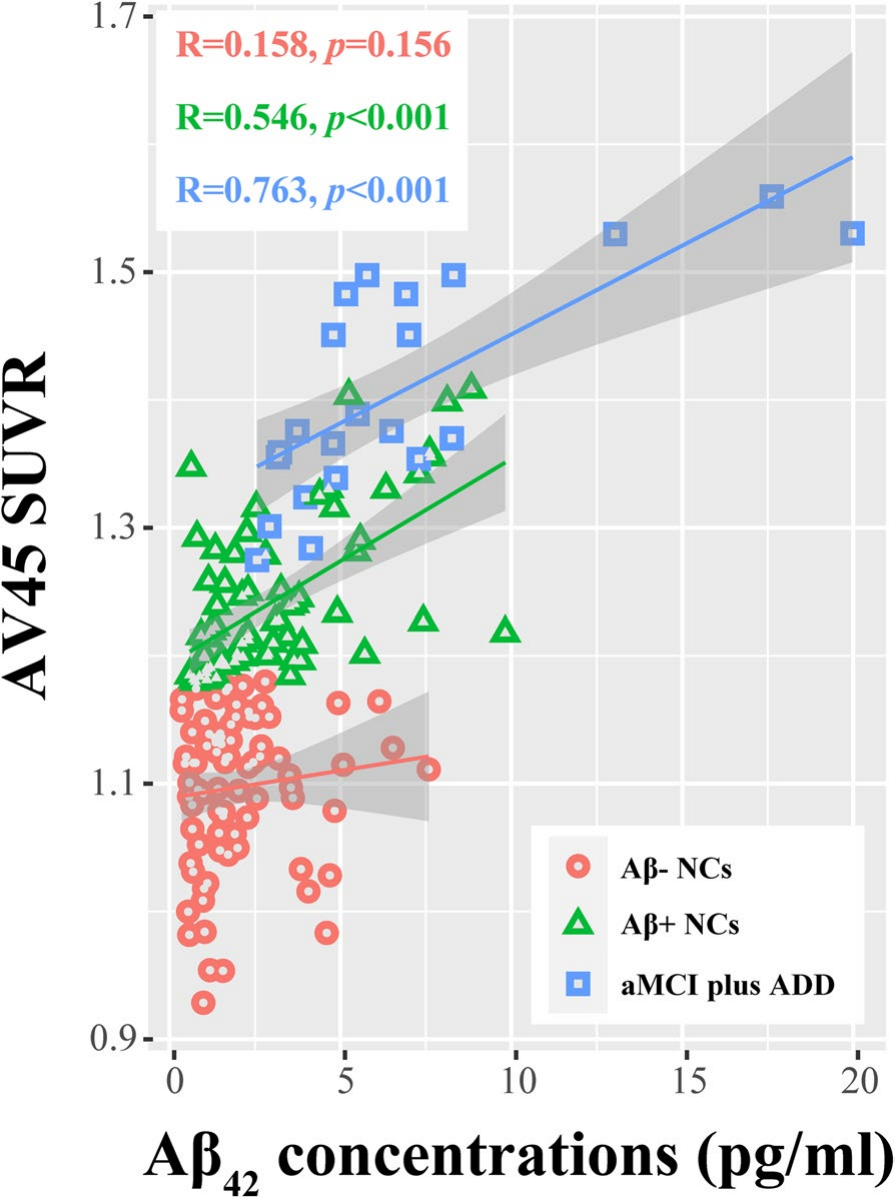
Relationship between nEV Aβ_42_ levels and AV45 SUVR. Correlation analysis was performed between the nEV Aβ_42_ levels and the AV45 SUVR in the different groups; the correlation coefficients and *p* values are listed. Aβ, β-amyloid; NCs, cognitively normal controls; aMCI, amnestic mild cognitive impairment; ADD, Alzheimer’s disease dementia; nEV, neuronal-derived extracellular vesicle; AV45, [18F]florbetapir; SUVR, standardized uptake value ratio

Importantly, nEV Aβ_42_ significantly and positively correlated with the AV45 SUVR in both the total cohort (*R* = 0.532, *p* < 0.001) and the subgroups (except in Aβ− NCs; Fig. 3). Linear regression analysis suggested that the nEV Aβ_42_ levels alone explained 41.1% of the variation in the average AV45 uptake. The addition of clinical features increased this level to 46.4% and did not affect the contribution of Aβ_42_ (*p* < 0.001). Additionally, there was no interaction between Aβ_42_ and *APOE* genotype (Table 3). Focusing on the NC groups did not affect these results (Additional file 1: Table S6). Furthermore, we explored the relationship between regional AV45 SUVR and nEV Aβ_42_ concentrations. In the total cohort, there was a significant positive association in all regions investigated even after correction for multiple comparisons analysis, except for the bilateral hippocampus (Table 4). Similar results were obtained in Aβ+ NCs, but not in Aβ− NCs or in patients with cognitive impairment. All analyses were adjusted for confounding factors.

**Table 3:** Relationships between nEV Aβ_42_ and AV45 SUVR

	***β***	Standard error	Standard ***β***	***t***	***p***
**The plasma nEV Aβ**_**42**_ **term alone explained 41.1% variation in average AV45 uptake**					
**Intercept**	1.109	0.011		104.429	**< 0.001**
**nEV Aβ**_**42**_	0.028	0.003	0.641	10.921	**< 0.001**
**The plasma nEV Aβ**_**42**_ **plus clinical features explained 46.4% variation in average AV45 uptake**					
**Intercept**	0.939	0.071		13.225	**< 0.001**
**nEV Aβ**_**42**_	0.025	0.003	0.573	9.695	**< 0.001**
**Age**	0.003	0.001	0.154	2.691	**0.008**
**Female**	− 0.011	0.015	− 0.043	− 0.763	0.447
***APOE*** **ε4 status**	0.041	0.015	0.165	2.819	**0.005**
**The plasma nEV Aβ**_**42**_ **plus clinical features and the interaction term explained 46.6% variation in average AV45 uptake**					
**Intercept**	0.915	0.077		11.986	**< 0.001**
**nEV Aβ**_**42**_	0.029	0.005	0.651	5.986	**< 0.001**
**Age**	0.003	0.001	0.166	2.815	**0.005**
**Female**	− 0.01	0.015	− 0.039	− 0.691	0.491
***APOE*** **ε4 status**	0.055	0.022	0.221	2.517	**0.013**
**nEV Aβ**_**42**_ * ***APOE*** **ε4 status**	− 0.005	0.006	− 0.121	− 0.858	0.392

The analysis was performed in total individuals including Aβ− NCs, Aβ+ NCs, and patients with aMCI and ADD. In the first model, nEV Aβ_42_ was used as a predictor of AV45 SUVR; in the second model, nEV Aβ_42_ plus age, sex, and *APOE* ε4 status were used as predictors of AV45 SUVR; in the third model, the interaction term between nEV Aβ_42_ and *APOE* ε4 status was additionally included

*Abbreviations*: *Aβ* β-amyloid, *NCs* cognitively normal controls, *aMCI* amnestic mild cognitive impairment, *ADD* Alzheimer’s disease dementia, *APOE* apolipoprotein E, *nEV* neuronal-derived extracellular vesicle, *AV45* [^18^F]florbetapir

**Table 4:** Relationships between nEV Aβ_42_ and regional AV45 SUVR

		Total participants	Aβ− NCs	Aβ+ NCs	aMCI plus ADD
**L_temporal lobe**	**R**	0.4618	0.0490	0.4178	0.2751
	***p***	**0.0000**	0.6678	**0.0005**	0.2544
**R_temporal lobe**	**R**	0.5493	0.0770	0.5383	0.5156
	***p***	**0.0000**	0.5000	**0.0000**	**0.0239**
**L_parietal lobe**	**R**	0.5699	0.0332	0.4662	0.6309
	***p***	**0.0000**	0.7715	**0.0001**	**0.0038**
**R_parietal lobe**	**R**	0.6029	0.1266	0.4573	0.7577
	***p***	**0.0000**	0.2662	**0.0001**	**0.0002**
**L_frontal lobe**	**R**	0.5808	0.0815	0.5514	0.7901
	***p***	**0.0000**	0.4751	**0.0000**	**0.0001**
**R_frontal lobe**	**R**	0.6043	0.1555	0.5214	0.7979
	***p***	**0.0000**	0.1713	**0.0000**	**0.0000**
**L_Pre**	**R**	0.5492	− 0.0356	0.4839	0.4321
	***p***	**0.0000**	0.7558	**0.0000**	0.0647
**R_Pre**	**R**	0.5359	− 0.0051	0.4701	0.4264
	***p***	**0.0000**	0.9645	**0.0001**	0.0687
**L_ACC**	**R**	0.5396	− 0.0318	0.5702	0.2565
	***p***	**0.0000**	0.7812	**0.0000**	0.2891
**R_ACC**	**R**	0.5074	0.0570	0.5480	0.3642
	***p***	**0.0000**	0.6181	**0.0000**	0.1253
**L_PCC**	**R**	0.4744	0.0901	0.4891	0.3325
	***p***	**0.0000**	0.4299	**0.0000**	0.1643
**R_PCC**	**R**	0.2522	0.0292	0.0430	0.2076
	***p***	**0.0009**	0.7983	0.7317	0.3938
**L_Ent**	**R**	0.4441	− 0.0206	0.3807	0.3183
	***p***	**0.0000**	0.8571	**0.0016**	0.1841
**R_Ent**	**R**	0.4927	0.0240	0.4267	0.5008
	***p***	**0.0000**	0.8334	**0.0004**	0.0290
**L_HP**	**R**	0.0344	0.0007	− 0.2287	0.2958
	***p***	0.6563	0.9953	0.0647	0.2188
**R_HP**	**R**	0.0599	− 0.0455	− 0.1864	0.2974
	***p***	0.4377	0.6904	0.1339	0.2162

The partial correlation analyses between nEV Aβ_42_ and regional AV45 SUVR were performed in all participants (*n* = 179), Aβ− NCs (*n* = 84), Aβ+ NCs (*n* = 72), and aMCI plus ADD individuals (*n* = 23), with correction for age, sex, and *APOE* ε4 status; the correlation coefficients and *p* values are listed

*Abbreviations*: *Aβ* β-amyloid, *NCs* cognitively normal controls, *aMCI* amnestic mild cognitive impairment, *ADD* Alzheimer’s disease dementia, *APOE* apolipoprotein E, *nEV* neuronal-derived extracellular vesicle, *AV45* [^18^F]florbetapir, *SUVR* standardized uptake value ratio, *Pre* precuneus, *ACC* anterior cingulate cortex, *PCC* posterior cingulate cortex, *Ent* entorhinal cortex, *HP* hippocampus

### Relationships with cognitive decline and brain atrophy

**Fig. 4:**
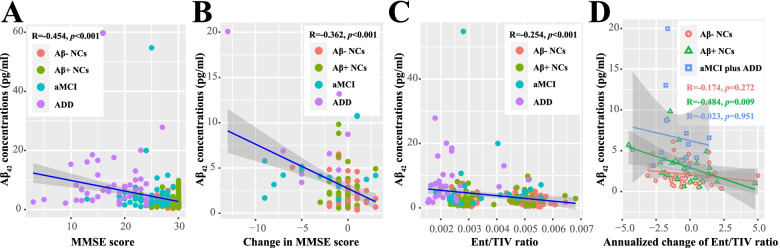
Associations of nEV Aβ_42_ concentrations with cognitive scales and brain regional volume. Correlation analysis was performed between the nEV Aβ_42_ concentrations and baseline MMSE scores (**A**) and the nEV Aβ_42_ concentrations and baseline Ent volume (**C**). Volume analysis was performed in 192 subjects who had baseline sMRI assessments, including 75 Aβ− NCs, 62 Aβ+ NCs, 27 aMCI, and 28 ADD individuals. Volume was expressed as the ratio to TIV. **B** Plots show the subset who had follow-up cognitive assessments (104 subjects including 49 Aβ− NCs, 33 Aβ+ NCs, 14 aMCI, and 8 ADD, with an average follow-up time of 14.58 ± 6.37 months). Correlation analysis was performed between the nEV Aβ_42_ concentrations and longitudinal changes in MMSE scores. **D** Correlation analysis was performed between the baseline nEV Aβ_42_ concentrations and longitudinal changes in Ent volume. The longitudinal volume changes were annualized and expressed as the ratio to TIV (multiplied by 10^4^), and the analyses were performed in 88 subjects who had follow-up sMRI assessments, including 43 Aβ− NCs, 31 Aβ+ NCs, 11 aMCI, and 3 ADD individuals, at an average follow-up time of 13.26 ± 4.7 months. The correlation coefficients and *p* values are listed. Aβ, β-amyloid; NCs, cognitively normal controls; aMCI, amnestic mild cognitive impairment; ADD, Alzheimer’s disease dementia; nEV, neuronal-derived extracellular vesicle; MMSE, mini-mental state examination; Ent, entorhinal cortex; TIV, total intracranial volume; sMRI, structural magnetic resonance imaging

A subset of individuals (*n* = 104) had a follow-up cognitive assessment after 14.58 ± 6.37 months. As shown, nEV Aβ_42_ levels correlated with both baseline scores (*p* < 0.001) and longitudinal worsening (*p* < 0.001) in MMSE (Fig. 4) and MoCA-B (Additional file 1: Fig. S7). After correcting for age, sex, and *APOE* ε4 genotype, the results remained statistically significant (Additional file 1: Table S7; also with analysis by diagnostic group).

We further analyzed the correlation between nEV Aβ_42_ and regional brain volumes. Specifically, cross-sectional analyses revealed that the index was significantly correlated with the volumes of the entorhinal cortex (Fig. 4C), hippocampus, and GM but not with those of the PCC and precuneus (Additional file 1: Fig. S8). After correcting for confounding factors, results remained statistically significant only for the entorhinal cortex (*p* = 0.039; Additional file 1: Table S8). A subset of individuals (*n* = 88) had a follow-up sMRI after 13.26 ± 4.7 months. The nEV Aβ_42_ levels correlated with longitudinal changes in the volumes of the entorhinal cortex and GM, but not that of other regions (Additional file 1: Fig. S9). After correcting for confounding factors, the results remained statistically significant only in the entorhinal cortex (*p* = 0.013; Additional file 1: Table S8). Notably, the correlation was only significant in Aβ+ NCs (*p* = 0.009, or 0.034 after correcting for confounding factors), but not in Aβ− NCs or in cognitively impaired patients (Fig. 4D and Additional file 1: Table S8; also with analysis by diagnostic group). Considering the changes as percentages did not affect the results.

## Discussion

Using a Chinese community-based population, we analyzed the AD-related cargos of EVs and found gradually increasing concentrations of Aβ_42_ along the Alzheimer’s continuum (from Aβ− NCs, through Aβ+ NCs, aMCI, to ADD). In contrast, the plasma Aβ concentrations did not change among the groups. More specifically, our study verified previous findings that the nEV Aβ_42_ assay indeed provides high diagnostic accuracy in identifying patients with cognitive impairment [9, 13]. Moreover, our study attempted to add new evidence for the preclinical stage of AD [12] and proved that the concentration of nEV Aβ_42_ is already increased in Aβ+ NCs, although its diagnostic efficacy was not marked (AUC with *APOE* genotype = 0.705). Furthermore, nEV Aβ_42_ levels were strongly associated with global and regional AV45 SUVR, suggesting that they reflect brain Aβ deposition. In addition, baseline nEV Aβ_42_ levels predicted longitudinal changes in cognition and entorhinal volume.

The specific enrichment of nEVs using immunoprecipitation is a pioneering discovery [30], opening a “window into the brain.” The blood-isolated total EVs are derived from a wide range of sources and cannot specifically reflect the changes in neuronal function, unlike the CD171+ EVs. Enrichment of CD171+ EVs of neuronal origin is mainly based on the fact that they contain higher levels of multiple neuronal markers, as shown in our study and previous studies [12, 31]. Our isolation protocol and its validation followed the MISEV2018 recommendations [25], further verifying the reliability. However, the quality control results in this study were unexpected. Neither p-tau181 nor markers of neuronal damage, including t-tau (in the pre-experiment) and NFL, were included in the subsequent analysis. Theoretically, compared to the traditional enzyme-linked immunosorbent assay (ELISA) method used in previous studies [8, 9, 11, 13], electrochemiluminescence and Simoa immunoassays are more sensitive. In addition, although the electrochemiluminescence method was fully suitable for the detection of plasma Aβ, its sensitivity was not sufficient to detect nEV Aβ (in the pre-experiment), which also violates the previous findings in which nEV Aβ could be quantified using ELISA [9, 13]. Fortunately, consistent with a recent study [12], the Simoa method reliably detected Aβ in nEVs. Several factors may account for the differences between our study and previous studies. First, there were differences in plasma volume, experimental procedures, and quality controls among studies. Second, besides the differences in assay platforms, different kits may be equipped with different antibody pairs. Additionally, the NFL detection kit was newly developed, and to the best of our knowledge, this kit and the p-tau181 detection kit were applied to nEVs here for the first time, thus requiring further verification. Third, it should be noted that some researchers have found that the nEV t-tau was largely undetectable using ELISA or Luminex immunoassays and proposed that previously reported quantifications may have resulted from contamination [32]. Considering our electrochemiluminescence results of nEV t-tau, we consider that its concentrations are probably too low to be detected. P-tau181 is derived from t-tau [33]; thus, it may be reasonable to speculate that nEV p-tau181 is also undetectable.

The ability of plasma Aβ to assist in AD diagnoses has been questioned over the years [10, 34]. The results are easily influenced by detection platforms [35], and blood Aβ is not necessarily brain-derived [36], making it difficult for it to replace CSF Aβ as a reliable biomarker. The electrochemiluminescence method has been reliably applied to the detection of CSF Aβ [37]; however, our study found that plasma Aβ had limited roles in the diagnosis of AD patients, let alone those in the preclinical stage, and it was not correlated with amyloid-PET results. In comparison, nEV Aβ_42_ can distinguish cognitively impaired patients from NCs. However, we admit that its ability to recognize Aβ+ NCs is not outstanding. According to our recent study on the discrimination of Aβ+ NCs [16], we believe that the values are reasonable because large-scale neurodegeneration has not yet occurred, and this stage represents the earliest identifiable preclinical stage [38]. Our study partially proved previous conclusions that nEV Aβ_42_ can predict MCI conversion [39] and that its concentrations increased with disease progression [9]. However, a recent study found that the assay was not helpful for the construction of a diagnostic model of preclinical AD, and the reasons for this may be complex [12]. We found that the nEV Aβ_40_ and Aβ_42_ were closely correlated, which was contrary to a previous study on nEV Aβ_40_ [40] and our viewpoint about their ratio. The reasons for this remain unclear after discussion with Quanterix™. Currently, EV normalization methods are not unified; either surface markers [13] or particle numbers can be used [12]. Recent findings suggest that the surface markers that are typically used are present in only a fraction of EVs and are not particularly enriched in smaller EVs in the exosome range [41]; therefore, normalization by particle number is likely to be more accurate.

The strong correlations between baseline nEV Aβ_42_ and AV45 SUVR indicated that nEVs have the potential to reflect brain pathological changes and are emerging as liquid biopsy tools. Furthermore, nEV Aβ_42_ was also associated with cognitive deterioration, as well as with entorhinal atrophy, suggesting that the blood assay could also serve as a predictor of disease progression, and thus could be used to select individuals most likely to progress during typically short clinical trial periods. We analyzed multiple brain regions; however, only the entorhinal cortex remained significant after correcting for confounding factors. The entorhinal cortex region is characteristically affected by tau pathology at an early stage in AD [1]. Compared to NCs, individuals with subtle cognitive difficulties demonstrate faster atrophy of the entorhinal cortex [42], and the volume, glucose metabolism, blood flow, and texture features of this region all play roles in predicting cognitive decline [24, 43–45]. Subgroup analysis suggested that the correlation between nEV Aβ_42_ and entorhinal atrophy only existed in Aβ+ NCs, but not in Aβ− NCs, indicating that the more severe the pathological damage, the more severe the atrophy. However, elevated brain Aβ deposition alone is probably insufficient to produce neuronal damage and cognitive changes [46], and their associations are likely mediated by neurofibrillary tangles, with a temporal delay [47, 48]. Nevertheless, based on the “amyloid cascade hypothesis” or real-world studies [1, 49], Aβ is the actual initiating factor of downstream pathological changes in AD. The dual effects of Aβ and tau aggravated the deterioration of cognition more than tau alone [50], and Aβ is an independent risk factor for cognitive impairment [51].

### Limitations

This study has several limitations. First, the small sample size limited the statistical power of our data, and not all participants underwent amyloid-PET. Second, CD171 is not absolutely expressed in the brain, but also in other tissues, and CD171+ EVs are thus not absolutely of neuronal origin only [31]. Third, longitudinal follow-up data are still lacking, and the follow-up time varied significantly. Fourth, due to the lack of tau pathology, it is unknown whether the relationship between nEV Aβ and neurodegeneration is mediated by tau. Fifth, extracting nEVs and quantifying their cargo are complex and expensive, and NTA tests are time-consuming. These factors limit their clinical application. Sixth, there is a lack of cognitively impaired patients with other neurodegenerative diseases. Seventh, recent findings have suggested that, although plasma Aβ detected by the Simoa method could not predict amyloid status among NCs well, it has a certain value in the diagnosis of symptomatic AD [29, 35, 52–54]. In the future, we will use Simoa assays instead of the electrochemiluminescence method to detect plasma Aβ for a matched and more meaningful horizontal comparison of nEV Aβ. Recently, a series of studies have suggested that phosphorylated tau proteins in the blood are reliable biomarkers of AD [10]. Their diagnostic effects have been verified in the Alzheimer’s continuum from multiple perspectives, including CSF, PET, autopsy, and clinical follow-ups. This weakens the significance of our study to a certain extent. However, blood tau is less stable than Aβ [55]. Additionally, EVs are essential for intercellular communication [6]. Thus, the extraction, validation, and cargo detection of nEVs may provide a basis for subsequent functional studies [56, 57]. Considering the shortcomings of our research and the limitations in this field, multicenter collaboration to include more pathology-identified participants is required in the future.

## Conclusions

In conclusion, our findings suggest that plasma nEV Aβ_42_ contributes to the diagnosis of AD-induced cognitive impairment and also discriminates Aβ+ NCs from Aβ− NCs. This blood biomarker reflects cortical amyloid deposition and predicts cognitive decline and entorhinal atrophy. Our findings highlight the clinical utility of nEV Aβ_42_ in identifying at-risk individuals and conducting disease-modifying trials.

## Supplementary Information

**Additional file 1. **Supplementary material. **Supplementary Table 1.** Summary of previous studies on Aβ, tau, and p-tau (detected in the plasma nEV) as biomarkers of AD. **Supplementary Table 2.** Quality control of the nEV protein. **Supplementary Table 3.** Baseline characteristics of all subjects with plasma Aβ levels by clinical diagnosis. **Supplementary Table 4.** Comparisons between participants with and without sMRI or scales data. **Supplementary Table 5.** Relationships between nEV Aβ_42_ and age, sex, group, and *APOE* ε4 status. **Supplementary Table 6.** Relationship between nEV Aβ_42_ and AV45 SUVR. **Supplementary Table 7.** Relationship between baseline nEV Aβ_42_ and cognitive scales. **Supplementary Table 8.** Relationship between baseline nEV Aβ_42_ and brain regional volume. **Supplementary Figure 1.** Typical TEM images of nEV. **Supplementary Figure 2.** Typical NTA results. **Supplementary Figure 3.** NTA results of enrolled subjects. **Supplementary Figure 4.** Western blot characterization of nEVs. **Supplementary Figure 5.** nEV Aβ concentrations in different diagnostic groups and the ROC curves. **Supplementary Figure 6.** Plasma Aβ concentrations in different diagnostic groups.**Supplementary Figure 7.** Association between nEV Aβ_42_ concentrations and MoCA-B scale. **Supplementary Figure 8.** Association between nEV Aβ_42_ concentrations and baseline brain regional volume. **Supplementary Figure 9.** Association between nEV Aβ_42_ concentrations and longitudinal changes in brain regional volume.

## Data Availability

The datasets used and/or analyzed during the current study are available from the corresponding authors on reasonable request.

## Abbreviations

AD: Alzheimer’s disease
Aβ: Amyloid-β
CSF: Cerebrospinal fluid
PET: Positron emission tomography
EVs: Extracellular vesicles
nEVs: Neuronal-derived EVs
p-tau181: Tau phosphorylated at threonine 181
sMRI: Structural magnetic resonance imaging
NCs: Cognitively normal controls
SILCODE: Sino Longitudinal Study on Cognitive Decline
APOE: Apolipoprotein E
AVLT: Auditory verbal learning test
AFT: Animal fluency test
BNT: Boston naming test
STT: Shape trails test
MMSE: Mini-Mental State Examination
MoCA-B: Montreal Cognitive Assessment-Basic
CDR: Clinical Dementia Rating Scale
MCI: Mild cognitive impairment
aMCI: Amnestic MCI
ADD: AD dementia
AV45: [^18^F]florbetapir
SUVR: Standardized uptake value ratio
SD: Standard deviation
PCC: Posterior cingulate cortex
TIV: Total intracranial volume
GM: Gray matter
TEM: Electron microscopy
NTA: Nanoparticle tracking analysis
NFL: Neurofilament light
AUC: Area under the curve
CI: Confidence interval
ROC: Receiver operating characteristic
