# Supplementary material for: β-Amyloid in blood neuronal-derived extracellular vesicles is elevated in cognitively normal adults at risk of Alzheimer’s disease and predicts cerebral amyloidosis
Source: Alzheimers Res Ther. 2022 May 12;14:66. doi: 10.1186/s13195-022-01010-x (PMC9097146; doi:10.1186/s13195-022-01010-x)
Supplement: Supplementary file 1 — Additional file 1. Supplementary material. Supplementary Table 1. Summary of previous studies on Aβ, tau, and p-tau (detected in the plasma nEV) as biomarkers of AD. Supplementary Table 2. Quality control of the nEV protein. Supplementary Table 3. Baseline characteristics of all subjects with plasma Aβ levels by clinical diagnosis. Supplementary Table 4. Comparisons between participants with and without sMRI or scales data. Supplementary Table 5. Relationships between nEV Aβ42 and age, sex, group, and APOE ε4 status. Supplementary Table 6. Relationship between nEV Aβ42 and AV45 SUVR. Supplementary Table 7. Relationship between baseline nEV Aβ42 and cognitive scales. Supplementary Table 8. Relationship between baseline nEV Aβ42 and brain regional volume. Supplementary Figure 1. Typical TEM images of nEV. Supplementary Figure 2. Typical NTA results. Supplementary Figure 3. NTA results of enrolled subjects. Supplementary Figure 4. Western blot characterization of nEVs. Supplementary Figure 5. nEV Aβ concentrations in different diagnostic groups and the ROC curves. Supplementary Figure 6. Plasma Aβ concentrations in different diagnostic groups.Supplementary Figure 7. Association between nEV Aβ42 concentrations and MoCA-B scale. Supplementary Figure 8. Association between nEV Aβ42 concentrations and baseline brain regional volume. Supplementary Figure 9. Association between nEV Aβ42 concentrations and longitudinal changes in brain regional volume. [file 13195_2022_1010_MOESM1_ESM.docx]

**Imaging acquisition**

Our previous studies described the imaging acquisition protocol and processing steps clearly [1-5]. Briefly, before undergoing imaging, subjects were instructed to keep their eyes closed but not fall asleep, relax their minds, and move as little as possible during imaging. Foam pads and headphones were used to minimize head movement and scanner noise. The T1-weighted images were acquired with a magnetization-prepared rapid gradient echo sequence: field of view (FOV) = 256×256 mm^2^, matrix = 256×256, slice thickness = 1 mm, gap = 0, slice number = 192, repetition time (TR) = 6.9 ms, echo time (TE) = 2.98 ms, inversion time = 450 ms, flip angle = 12^°^, voxel size = 1×1×1 mm^3^; the amyloid-positron emission tomography (PET) images were acquired 40 minutes after intravenous injection of ^18^F-AV45 (^18^F-florbetapir; 259-370 MBq), and data were recorded using a time-of-flight ordered subset expectation maximization algorithm with the following parameters: scan duration = 35 min, eight iterations, 32 subsets matrix = 192×192, FOV = 350×350, half-width height = 3.

**Amyloid-PET processing**

The processing steps were as follows. First, the original DICOM data were converted to the NIfTI file format using DCM2NII (<https://people.cas.sc.edu/rorden/mricron/dcm2nii.html>). Second, the T1-weighted images were segmented using the CAT12 toolbox (<http://dbm.neuro.uni-jena.de/cat/>). Third, the PET images were co-registered with their corresponding gray matter (GM) images and corrected for partial volume effect (PVE) using the PETPVE12 toolbox ([http://www.fil.ion.ucl.ac.uk/spm/ext/#PETPVE12](http://www.fil.ion.ucl.ac.uk/spm/ext/%23PETPVE12)), which is based on the Muller-Gartner algorithm, in order to provide a better approximation of the true regional tracer uptake [6]. Fourth, the GM images were normalized to the Montreal Neurological Institute (MNI) standard space, and the PVE-corrected PET images were normalized to the MNI space using the forward transformation parameters determined by the GM image spatial normalization. Finally, an 8-mm full width at half-maximum (FWHM) Gaussian kernel was used to smooth the images. All procedures were implemented using the Statistical Parametric Mapping (SPM12) software ([www.fil.ion.ac.uk/spm](http://www.fil.ion.ac.uk/spm)). For amyloid-PET, with reference to our previous studies [1-4], the whole cerebellum was used as the reference region and the whole cerebral cortex was used as the region of interest to calculate the standardized uptake value ratio (SUVR). In addition to the global index, we also obtained brain regional AV45 SUVR including the temporal lobe, parietal lobe, frontal lobe, precuneus, anterior cingulate cortex, posterior cingulate cortex, entorhinal cortex, and hippocampus. These regions are cortical areas or typical areas with early AD-related pathological proteins deposition and neurodegeneration [7].

**Brain sMRI processing**

Previous studies have verified that CAT12 is an advanced and computationally efficient brain segmentation tool that can provide accurate volumetric analysis and is consistent with FreeSurfer [8-11]. Details of the calculation procedures have been described in <http://www.neuro.uni-jena.de/cat12/CAT12-Manual.pdf>. For structural magnetic resonance imaging (sMRI), region-of-interest (ROI)-based morphometry analysis was performed in the following steps. First, the original DICOM data were converted to the NIfTI file format. Second, the images were normalized and showed a spatial resolution of 1.5 mm × 1.5 mm × 1.5 mm voxel size after being segmented into GM, white matter, and cerebrospinal fluid tissues. Third, a modulation process was performed to compensate for the volume. Fourth, an ROI analysis based on the “neuromorphometrics” atlas was performed. After preprocessing and visual checks for artifacts, all scans were passed through an automated quality check. The results were stored as XML files in the label directory and included the volume data of 142 regions. The total intracranial volume (TIV) and GM volume were also obtained.

**Isolation of nEVs from plasma**

To isolate neuronal-derived extracellular vesicles (nEVs) from plasma, 250 μL plasma was incubated with thromboplastin-D (System Biosciences, Inc., CA) for 1 h after centrifugation (12000 × g, 20 min, 4°C), followed by 175 μL calcium- and magnesium-free Dulbecco’s balanced salt solution (Gibco™, USA), with protease (Roche Applied Sciences, Inc., USA) and phosphatase inhibitor cocktails (Thermo Scientific™, USA). After centrifugation (1500 × g, 20 min, 4°C), the supernatants were incubated with 126 μL of Exoquick® (System Biosciences, Inc., CA) for 1 h at 4°C. The resultant suspensions were centrifuged at 1500 × g for 20 min at 4°C to obtain pellets containing total extracellular vesicles (EVs). Next, the EVs were dissolved in ultrapure water with a protease inhibitor and then incubated with 4 μL mouse anti-human CD171 biotinylated antibody (Thermo Scientific™, USA) in 50 μL 3% bovine serum albumin for 2 h at room temperature, followed by the addition of 12.5 μL Streptavidin UltraLink resin (Thermo Scientific™, USA) in 50 μL phosphate buffer (Gibco™, USA) and further incubation on a rotational mixer with continuous gentle mixing for 1 h. After centrifugation (400 × g, 10 min, 4°C), the pellets were resuspended in 100 μL of 0.05 M glycine-HCl (pH = 3.0), vigorously mixed, incubated for 10 min at room temperature, and centrifuged at 3000 × g for 10 min at 4°C. The nEV-containing supernatant was transferred to a new Eppendorf tube and the pH adjusted to 7.5 with 1 M Tris-HCl. Finally, the supernatant was aliquoted for various applications. Tubes for immunoassay received M-PER mammalian protein extraction reagent (Thermo Scientific™, USA) containing protease and phosphatase inhibitors, underwent three freeze‒thaw cycles, and were then stored at -80°C.

**nEV characterizations**

The nEVs were observed on two different transmission electron microscopy (TEM) platforms: JEM-1400plus (JEOL, Akishima, Japan) from Capital Medical University and Tecnai Spirit (FEI, Hillsboro, OR, USA) from Tsinghua University. The fresh nEV solution was diluted with phosphate buffer and negatively stained. Briefly, the copper grids (Zhongjingkeyi Technology Co., Ltd., CN) were hydrophilized using a plasma cleaner (Harrick PDC-32G-2 Basic, Gatan Solarus, USA), after which the samples were stained with 3% uranyl acetate using the pendant drop method (30 s, twice). The surplus liquid was blotted dry using a Whatman filter paper. The EVs were observed under a pressure of 100 kV or 120 kV.

We performed nanoparticle tracking analysis (NTA) in all nEV preparations in this study, which is a painstaking process. Identical to previous findings [12], we found that NTA was easily influenced by setting parameters, such as the camera level (**data not shown**). Therefore, the analysis was performed by the same operator under the same parameters on the same platform for a concentrated period of time. Prior to lysis, 10 μL were taken from the nEV suspension, diluted in 1000 μL filtered phosphate buffer (filtered through a 0.22-μm filter membrane). The solutions were stored at -80°C before centralized detection. The instrument (NanoSight NS300; Peking University Health Science Center) was equipped with a CMOS camera (Hamamatsu Photonics, Hamamatsu, Japan). A 405-nm laser was used. Data acquisition and processing were performed using NTA software 3.3. Three individual 60-s measurements were recorded for each sample with automated analysis settings for blur, track length, and minimum expected particle size. The software automatically calculated the concentration and average diameter of nEVs.

To confirm that nEVs carry typical EV markers, we performed western blotting (WB) of nEVs and EV-depleted plasma (negative control) from four participants (**Supplementary Figure 4**). The EV-depleted plasma was the supernatant obtained after removing the EV pellet after Exoquick®. The protein content was determined using a BCA protein assay kit (Enhanced BCA Protein Assay Kit, Beyotime, CN). The samples were subjected to 10% sodium dodecyl sulfate‒polyacrylamide gel electrophoresis (P0456S, Beyotime, CN). After blotting onto a polyvinylidene difluoride membrane, the proteins were incubated at 4°C overnight with specific primary antibodies: anti-CD63 (1:200, sc-5275, santa cluz, USA), anti-TSG101 (1:5000, 67381-1-Ig, proteintech, CN), anti-Albumin (1:10000, 66051-1-Ig, proteintech, CN), anti-Tubb3 (1:1000, bs-2670R, Bioss, CN), anti-GM130 (1:2000, 11308-1-AP, proteintech, CN), and anti-SNAP-25 (1:1000, 14903-1-AP, proteintech, CN). After washing, the membrane was incubated with the corresponding horseradish peroxidase-coupled secondary antibody (AmyJet Scientific Inc., CN) and visualized by enhanced chemiluminescence (Millipore, USA) using a chemiluminescence gel imaging system (FluorChemHD2, ProteinSimple, USA). Notably, although previous studies have suggested the neuronal origin of EVs extracted using this method [13, 14], we further proved this.

**Quality control of plasma β-amyloid (A****β) quantification**

To assess the plate-to-plate variability for all assays, we used the kits of the same lot number to ensure comparability between the plates. In addition, we used homemade controls in the plates and found that the coefficient of variation (CV) value between plates was less than 20%. The experiment was completed by an experienced operator according to the manufacturer’s protocol. The lower limit of detection (LOD) refers to the data provided by the kit, which was defined as 22.2 pg/ml for Aβ_38_, 5.41 pg/ml for Aβ_40_, and 0.516 pg/ml for Aβ_42_. According to the manufacturer, the LOD is a calculated concentration corresponding to the signal 2.5 standard deviations above the background (zero calibrator), and the provided LODs were calculated based on 50 runs across three kit lots. The lower limit of quantification (LLOQ) was set as the calibrator point before the CV increased above 20%, and required a recovery rate between 80% and 120%. The LLOQ of each plate was calculated and the highest value was defined as the final LLOQ. Specifically, the LLOQ of Aβ_38_ was 57.81 pg/ml, that of Aβ_40_ was 14.84 pg/ml, and that of Aβ_42_ was 2.30 pg/ml.

We found that this kit was not suitable for plasma Aβ_38_. Specifically, the signal values of 47.6% of the subjects (218/458) were lower than the LOD, indicating that the concentrations could not be fitted. Therefore, we did not analyze the data. For plasma Aβ_40_, 13 samples had levels below the LLOQ. For plasma Aβ_42_, levels in 26 samples were below the LLOQ. If their values exceeded the LOD and CV < 20%, these samples were considered truly low and were assigned the LLOQ value (2/13 samples for Aβ_40_, 9/26 samples for Aβ_42_). If CV ≥ 20%, they were excluded from the analysis (0 samples). If their values were below the LOD, they were excluded from analysis.

**Quality control of nEV protein quantification**

To assess the plate-to-plate variability for all assays, we used kits of the same lot number to ensure comparability between plates (**Supplementary Table 2**). Furthermore, the concentrations of the positive controls provided by the manufacturer were within the effective range for each run. The two positive controls included a low-concentration standard and high-concentration standard. The experiment was completed by an experienced operator according to the manufacturer’s protocol. The LOD and LLOQ values were determined using the SIMOA 5PL Assay Developer Tool for Aβ_40_ and Aβ_42_, and the SIMOA 4PL Assay Developer Tool for neurofilament light (NFL) and tau phosphorylated at threonine 181 (p-tau181), as provided by the manufacturer. As shown in **Supplementary Table 2**, the values obtained in our study were similar to those provided by the manufacturer. The highest value of each run was defined as the final LOD or LLOQ. Specifically, the LOD of Aβ_40_ was 0.6290 pg/ml, that of Aβ_42_ was 0.1215 pg/ml, that of NFL was 0.0509 pg/ml, and that of p-tau181 was 0.0446 pg/ml. The LLOQ of Aβ_40_ was 1.1800 pg/ml, that of Aβ_42_ was 0.2300 pg/ml, that of NFL was 0.3990 pg/ml, and that of p-tau181 was 0.117 pg/ml.

We found that these kits were not suitable for assessing NFL and p-tau181 in nEVs. Specifically, the signal values of 89.8% of the subjects (221/246) were lower than the LLOQ (64/221 were lower than the LOD) for NFL, and those of 83.3% of the subjects (205/246) were lower than the LLOQ (110/205 were lower than the LOD) for p-tau181. Therefore, we did not analyze the data. To the best of our knowledge, no previous study had detected NFL in nEVs. The results of p-tau181 were in contrast to previous studies (**Supplementary Table 1**), as we have explained in the “**Discussion**” section. In 31 samples, nEV Aβ_40_ levels were below the LLOQ. For plasma Aβ_42_, levels were below the LLOQ in seven samples. If their values were above the LOD and CV < 20%, these samples were considered truly low and were assigned the LLOQ value (19/31 samples for Aβ_40_, 0 samples for Aβ_42_), whereas if CV ≥ 20%, they were excluded from the analysis (3/31 samples for Aβ_40_, 0 samples for Aβ_42_). If their values were below the LOD, they were excluded from analysis. In addition, in seven samples, levels were above the LLOQ value for Aβ_40_, but had CV ≥ 20%; these were also excluded from the analysis.

| **Supplementary Table 1. Summary of previous studies on Aβ, tau, and p-tau (detected in the plasma nEV) as biomarkers of AD.** | | | | | |
| --- | --- | --- | --- | --- | --- |
| **Samples** | **Biomarkers** | **Detection method** | **Normalization method** | **Significance** | **Reference** |
| Cross-sectional study: AD, 57 (aMCI, 29, CDR 0.5; dementia, 28, CDR 1.0); NC, 57 | Total tau, p-tau181, p-S396-tau, Aβ_42_ | ELISA | CD81 | Total-tau: AD = NC  P-tau181, p-S396-tau, Aβ_42_: AD > NC | [15] |
| Longitudinal study: clinical converters, 24, with 24 blood samples at preclinical stage (AP), 24 at the time of initial AD diagnosis (aMCI, 13; dementia, 11); NC, 24 |  |  |  | Total-tau: AP = AD = NC  P-tau181, p-S396-tau: (AP = AD) > NC  Aβ_42_: AD > AP > NC |  |
| AD, 12 (aMCI, CDR 0.5; dementia, CDR 1.0); NC, 10 | P-tau181, p-S396-tau, Aβ_42_ | ELISA | CD81 | P-tau181, p-S396-tau, Aβ_42_: AD > NC | [16] |
| Mild-to-moderate AD-dementia, 10; stable MCI, 20; unstable MCI, 20; NC, 10 | P-tau181, p-S396-tau, Aβ_42_ | ELISA | CD81 | P-S396-tau, Aβ_42_: (AD = unstable MCI) > (stable MCI = NC)  P-tau181: unstable MCI > (stable MCI = AD) > NC | [17] |
| Discovery cohort: AD-dementia, 28; aMCI, 25; NC, 29 | Total tau, p-tau181, Aβ_42_ | ELISA | CD81 | Total tau, p-tau181, Aβ_42_: AD > aMCI > NC  Significantly correlated with their corresponding CSF levels | [18] |
| Validation cohort: AD-dementia, 73; aMCI, 71; NC, 72 |  |  |  |  |  |
| Clinical converters, 128, with 304 blood samples at preclinical stage (AP); non-converters, 222, with 583 samples before the deadline of follow-up, as the NC group | Total-tau, p-tau231, p-tau181, Aβ_42_ | MSD platform for total-tau, p-tau231, p-tau181; Simoa platform for Aβ_42_ | Direct comparison; normalized by EV concentration and EV average diameter in the prediction models | P-tau231, p-tau181: AP > NC  Total-tau, Aβ_42_: AP = NC  Aβ_42_ was not included in the best model for AD prediction | [14] |
| AD-dementia, 106; NC, 106 | Total-tau | Simoa | Not mentioned, probably direct comparison | AD = NC | [19] |
| AD-dementia, 20 (mild, 10, CDR 0.5-1; moderate, 10, CDR 2); MCI, 10, CDR 0.5; NC, 10 | Full-length tau, mid-region tau, p-tau181 | ELISA | CD81 | No changes among groups | [20] |
| Mild AD-dementia 18; MCI, 29; NC, 23 | Total-tau, p-S202-tau, p-tau181, Aβ_42_ | ELISA | CD63 | Total-tau: AD > (MCI = NC)  P-S202-tau: AD > (MCI = NC)  P-S202-tau/total-tau: MCI > (NC = AD)  P-tau181: AD = MCI = NC  Aβ_42_: slightly greater in the AD group than in the MCI and NC groups, not reached significance  P-tau181/Aβ_42_: no changes | [21] |
| Among them, 19 subjects had cognitive deterioration 4 years later, 10 maintained the cognition |  |  |  | Total-tau, p-S202-tau: deterioration > maintain  P-tau181, Aβ_42_: deterioration = maintain |  |
| MCI, 61; NC, 76 | Aβ_42_, p-S396-tau | ELISA | CD81 | Aβ_42_: MCI > NC  p-S396-tau: MCI = NC | [22] |
| AD-dementia, 88; MCI, 87; NC, 80 | Aβ_42_, Aβ_40_ | ELISA | Total protein levels for normalization of the relative values for each sample (ELISA); CD63 | Aβ_42_: AD-dementia > MCI > NC  Aβ_40_: AD-dementia = MCI = NC  Aβ_42_/Aβ_40_: AD-dementia > (MCI = NC) | [23] |
| Among the MCI, 8 to AD-dementia within 2 years, 16 within 3 years |  |  |  | Logistic regression: Aβ_42_ contributed to the prediction of MCI conversion |  |
| Sub-clinical cognitive decliners, 73 (2 to MCI); matched stable individuals, 73 | P-tau231, p-tau181, total-tau | MSD | Normalized by EV concentration and EV average diameter in the regression models | Declining individuals showed higher p-tau231, p-tau181, and total-tau levels with older age (60 years old) compared to stable individuals  At age 60, declining compared to stable individuals showed higher annualized change marginally for p-tau181 (*p*=0.08)  Older declining compared to stable individuals had lesser increases marginally for p-tau181 (*p*=0.07)  All were included in the best model for declining prediction | [24] |
| Wild type mice, 15; 2xTg-AD mice, 4; 5xFAD mice, 9; 3xTg-AD mice, 15 | Aβ_42_, p-tau181, total-tau | MILLIPLEX® MAP Human Amyloid Beta and Tau Panel | Not mentioned, probably direct comparison | Total-tau: 3xTg-AD > WT mice, no changes among the other groups  p-tau181: 3xTg-AD > WT mice and 5xFAD mice, no changes among the other groups  Aβ_42_: 5xFAD mice > the other three groups  The total-tau, p-tau181 and Aβ_42_ levels were strongly and positively correlated with their brain depositions | [25] |

Abbreviations: Aβ, β-amyloid; p-tau, phosphorylated tau; nEV, neuronal-derived extracellular vesicle; AD, Alzheimer's disease; MCI, mild cognitive impairment; aMCI, amnestic MCI; CDR, Clinical Dementia Rating scale; NC, cognitively normal control; AP, AD at the preclinical stage; p-tau181, tau phosphorylated at threonine 181; p-S396-tau, tau phosphorylated at serine 396; ELISA, enzyme linked immunosorbent assay; p-tau231, tau phosphorylated at threonine 231; MSD, meso scale discovery; Simoa, single-molecular array; p-S202-tau, tau phosphorylated at serine 202.

| **Supplementary Table 2. Quality control of the nEV protein.** | | |
| --- | --- | --- |
| **Quality control of the products** | | |
| **Product** | Simoa™ Neuro 4-Plex E Kit; Simoa™ pTau-181 Advantage V2 Kit | |
| **Product number** | 103670; 103714 | |
| **Lot number** | 503105; 503008 | |
| **Platform** | HD-X | |
| **LOD** | **Provided by the manufacturer** | **Provided by our experiments** |
| **Aβ_42_** | 0.136 pg/mL; range 0.0601-0.204 pg/mL | 0.1115-0.1215 pg/mL |
| **Aβ_40_** | 0.384 pg/mL; range 0.189-0.531 pg/mL | 0.3818-0.6290 pg/mL |
| **NFL** | 0.090 pg/mL; range 0.016-0.152 pg/mL | 0.0473-0.0509 pg/mL |
| **p-tau181** | 0.028 pg/mL; range 0.019-0.052 pg/mL | 0.0436-0.0446 pg/ml |
| **LLOQ** | **Provided by the manufacturer** | **Provided by our experiments** |
| **Aβ_42_** | 0.378 pg/mL | 0.2170-0.2300 pg/ml |
| **Aβ_40_** | 1.02 pg/mL | 0.8940-1.1800 pg/ml |
| **NFL** | 0.400 pg/mL | 0.3660-0.3990 pg/ml |
| **p-tau181** | 0.085 pg/mL | 0.107-0.117 pg/ml |

Abbreviations: Simoa, single-molecular array; LOD, limit of detection; LLOQ, lower limit of quantification; Aβ, β-amyloid; NFL, neurofilament light; p-tau181, tau phosphorylated at threonine 181; nEV, neuronal-derived extracellular vesicle.

| **Supplementary Table 3. Baseline characteristics of all subjects with plasma Aβ levels by clinical diagnosis.** | | | |
| --- | --- | --- | --- |
| **Clinical diagnosis** | **NCs** | **aMCI** | **ADD** |
| **Number of participants** | 370 | 47 | 41 |
| **Male** | 131 (35.4%) | 26 (55.3%)^*^ | 17 (41.5%)^#^ |
| **Age (y)** | 65.5±6.1 | 69.5±7.0^**^ | 73.1±9.0^***^ |
| **Education** | 12.4±3.4 | 10.4±4.1^*^ | 9.6±5.0^**^ |
| **MMSE (out of 30)** | 28.6±1.6 | 25.0±3.5^***^ | 16.2±5.8^***^ |
| ***APOE* ε4 carries** | 87 (23.5%) | 18 (38.3%)^#^ | 25 (61.0%)^*^ |
| **Plasma Aβ_40_, pg/ml** | 759.3±169.9 | 815.4±175.9^#^ | 833.3±262.0^#^ |
| **Plasma Aβ_42_, pg/ml** | 12.1±9.1 | 11.5±4.6^#^ | 11.2±4.5^#^ |
| **Aβ_42_/Aβ_40_ ratio** | 0.016±0.010 | 0.014±0.005^#^ | 0.013±0.004^#^ |

Data were summarized as numbers (%) or as means ± standard deviations for categorical and continuous variables, respectively. Statistical analyses were conducted using the chi-square test for categorical variables and the Kruskal‒Wallis H test for continuous variables, followed by multiple post hoc comparisons (adjusted *p* value). Compared with the NCs: * *p* < 0.05; ** *p* < 0.01; *** *p* < 0.001; #, > 0.05. Notably, 180 subjects were further included in the current nEV study, including 126 NCs (81 Aβ- NCs and 45 Aβ+ NCs), 34 aMCI, and 20 ADD individuals.

Abbreviations: NCs, cognitively normal controls; aMCI, amnestic mild cognitive impairment; ADD, Alzheimer’s disease dementia; MMSE, mini-mental state examination; APOE, apolipoprotein E; Aβ, β-amyloid; nEV, neuronal-derived extracellular vesicle.

| **Supplementary Table 4. Comparisons between participants with and without sMRI or scales data.** | | | | | | |
| --- | --- | --- | --- | --- | --- | --- |
| **Diagnosis** | **With** **baseline sMRI (n)** | **Without baseline sMRI (n)** | **Age** | **Sex** | **Education** | ***APOE* ε4 carriers** |
| **Aβ- NCs** | 75 | 9 | 0.317 | 0.260 | **0.042** | 1.000 |
| **Aβ+ NCs** | 62 | 20 | 0.332 | 0.285 | 0.644 | 0.097 |
| **aMCI** | 27 | 18 | 0.972 | 0.393 | 0.896 | 0.807 |
| **ADD** | 28 | 17 | 0.823 | 0.384 | 0.117 | 0.848 |
|  | **With** **longitudinal sMRI (n)** | **Without longitudinal sMRI (n)** |  |  |  |  |
| **Aβ- NCs** | 43 | 41 | 0.099 | 0.758 | 0.305 | **0.039** |
| **Aβ+ NCs** | 31 | 41 | 0.362 | 0.239 | 0.597 | 0.070 |
| **aMCI + ADD** | 14 | 76 | 0.875 | 0.812 | 0.057 | 0.389 |
|  | **With** **longitudinal scales (n)** | **Without longitudinal scales (n)** |  |  |  |  |
| **Aβ- NCs** | 49 | 35 | 0.243 | 0.754 | 0.566 | 0.200 |
| **Aβ+ NCs** | 33 | 39 | 0.256 | 0.316 | 0.468 | 0.751 |
| **aMCI + ADD** | 22 | 68 | 0.819 | 0.109 | 0.187 | 0.467 |

Statistical analyses were conducted using the chi-square test or Fisher’s exact test for categorical variables, and the independent two sample t-test for continuous variables. *p* values are provided for each variable.

Abbreviations: Aβ, β-amyloid; NCs, cognitively normal controls; aMCI, amnestic mild cognitive impairment; ADD, Alzheimer’s disease dementia; APOE, apolipoprotein E; sMRI, structural magnetic resonance imaging.

| **Supplementary Table 5. Relationships between nEV Aβ_42_ and age, sex, group, and *APOE* ε4 status.** | | | | | |
| --- | --- | --- | --- | --- | --- |
|  | **β** | **Standard Error** | **Standard β** | **t** | ***p*** |
| **Intercept** | 1.655 | 1.781 |  | 0.929 | 0.354 |
| **Aβ+ NCs** | 1.009 | 0.318 | 0.261 | 3.169 | **0.002** |
| **Female** | 0.101 | 0.323 | 0.025 | 0.312 | 0.755 |
| **Age** | -0.014 | 0.026 | -0.044 | -0.543 | 0.588 |
| ***APOE* ε4 carriers** | 0.291 | 0.326 | 0.073 | 0.893 | 0.373 |

The analysis was performed in NCs including Aβ- NCs and Aβ+ NCs; age, sex, group status, and *APOE* ε4 status were used as predictors of nEV Aβ_42_.

Abbreviations: Aβ, β-amyloid; NCs, cognitively normal controls; APOE, apolipoprotein E; nEV, neuronal-derived extracellular vesicle.

| **Supplementary Table 6. Relationship between nEV Aβ_42_ and AV45 SUVR.** | | | | | |
| --- | --- | --- | --- | --- | --- |
|  | **β** | **Standard Error** | **Standard β** | **t** | ***p*** |
| **The plasma nEV Aβ_42_ term alone explained 19.1% variation in average AV45 uptake** | | | | | |
| **Intercept** | 1.111 | 0.011 |  | 98.892 | **<0.001** |
| **nEV Aβ_42_** | 0.021 | 0.004 | 0.436 | 5.921 | **<0.001** |
| **The plasma nEV Aβ_42_ plus clinical features explained 23.5% variation in average AV45 uptake** | | | | | |
| **Intercept** | 0.94 | 0.079 |  | 11.884 | **<0.001** |
| **nEV Aβ_42_** | 0.02 | 0.004 | 0.419 | 5.733 | **<0.001** |
| **Age** | 0.002 | 0.001 | 0.162 | 2.233 | **0.027** |
| **Female** | -0.001 | 0.014 | -0.004 | -0.061 | 0.951 |
| ***APOE* ε4 status** | 0.029 | 0.014 | 0.149 | 2.032 | **0.044** |
| **The plasma** **nEV Aβ_42_ plus clinical features and the interaction term explained 24.0% variation in average AV45 uptake** | | | | | |
| **Intercept** | 0.923 | 0.081 |  | 11.415 | **<0.001** |
| **nEV Aβ_42_** | 0.023 | 0.005 | 0.481 | 5.023 | **<0.001** |
| **Age** | 0.003 | 0.001 | 0.171 | 2.338 | **0.021** |
| **Female** | <0.001 | 0.014 | -0.001 | -0.019 | 0.984 |
| ***APOE* ε4 status** | 0.048 | 0.024 | 0.247 | 2.022 | **0.045** |
| **nEV Aβ_42_ * *APOE* ε4 status** | -0.007 | 0.007 | -0.142 | -1.003 | 0.318 |

The analysis was performed in NCs including Aβ- NCs and Aβ+ NCs. In the first model, nEV Aβ_42_ was used as predictors of AV45 SUVR; in the second model, nEV Aβ_42_ plus age, sex, and *APOE* ε4 status were used as predictors of AV45 SUVR; in the third model, the interaction term between nEV Aβ_42_ and *APOE* ε4 status was additionally included.

Abbreviations: Aβ, β-amyloid; NCs, cognitively normal controls; APOE, apolipoprotein E; nEV, neuronal-derived extracellular vesicle; AV45, [^18^F]florbetapir; SUVR, standardized uptake value ratio.

| **Supplementary Table 7. Relationship between baseline nEV Aβ_42_ and cognitive scales.** | | | | | | | | | | |
| --- | --- | --- | --- | --- | --- | --- | --- | --- | --- | --- |
|  | **Baseline MMSE scores** | | | | | **Baseline MoCA-B scores** | | | | |
|  | **β** | **Standard Error** | **Standard β** | **t** | ***p*** | **β** | **Standard Error** | **Standard β** | **t** | ***p*** |
| **Among all participants** | -0.194 | 0.052 | -0.225 | -3.759 | **<0.001** | -0.248 | 0.058 | -0.242 | -4.306 | **<0.001** |
| **Among Aβ- NCs** | -0.455 | 0.13 | -0.397 | -3.512 | **<0.001** | -0.378 | 0.172 | -0.242 | -2.195 | **0.031** |
| **Among Aβ+ NCs** | 0.017 | 0.088 | 0.021 | 0.190 | 0.850 | -0.070 | 0.122 | -0.064 | -0.576 | 0.567 |
| **Among aMCI group** | 0.044 | 0.062 | 0.109 | 0.700 | 0.488 | -0.015 | 0.053 | -0.043 | -0.286 | 0.776 |
| **Among ADD group** | 0.032 | 0.077 | 0.058 | 0.408 | 0.685 | 0.080 | 0.066 | 0.178 | 1.216 | 0.231 |
|  | **Longitudinal changes of MMSE scores** | | | | | **Longitudinal changes of MoCA-B scores** | | | | |
|  | **β** | **Standard Error** | **Standard β** | **t** | ***p*** | **β** | **Standard Error** | **Standard β** | **t** | ***p*** |
| **Among all participants** | -0.360 | 0.084 | -0.394 | -4.287 | **0.000** | -0.337 | 0.096 | -0.350 | -3.512 | **0.000** |
| **Among Aβ- NCs** | -0.381 | 0.135 | -0.408 | -2.815 | **0.007** | -0.561 | 0.212 | -0.375 | -2.651 | **0.011** |
| **Among Aβ+ NCs** | 0.019 | 0.112 | 0.034 | 0.171 | 0.866 | -0.135 | 0.153 | -0.187 | -0.884 | 0.385 |
| **Among aMCI plus ADD groups** | -0.157 | 0.220 | -0.165 | -0.714 | 0.485 | -0.145 | 0.241 | -0.158 | -0.605 | 0.554 |

The analysis was performed in different groups with baseline MMSE (MoCA-B) scores or longitudinal changes of MMSE (MoCA-B) scores as dependent variable, nEV Aβ_42_ and age, sex, education, and *APOE* ε4 status as independent variables. The regression results of age, sex, education, and *APOE* ε4 status are not listed here. There were 104 subjects who had follow-up assessments of MMSE and MoCA-B, including 49 Aβ- NCs, 33 Aβ+ NCs, 14 aMCI and 8 ADD; the average follow-up time was 14.58 ± 6.37 months.

Abbreviations: MMSE, mini-mental state examination; MoCA-B, Montreal cognitive assessment-basic version; Aβ, β-amyloid; NCs, cognitively normal controls; aMCI, amnestic mild cognitive impairment; ADD, Alzheimer’s disease dementia; nEV, neuronal-derived extracellular vesicle; APOE, apolipoprotein E.

| **Supplementary Table 8. Relationship between baseline nEV Aβ_42_ and brain regional volume.** | | | | | | | | | | | | | | | |
| --- | --- | --- | --- | --- | --- | --- | --- | --- | --- | --- | --- | --- | --- | --- | --- |
|  | **Baseline Ent volume** | | | | | **Annualized change of Ent/TIV ratio** | | | | | **Annualized change of Ent volume (%)** | | | | |
|  | **β** | **SE** | **Standard β** | **t** | ***p*** | **β** | **SE** | **Standard β** | **t** | ***p*** | **β** | **SE** | **Standard β** | **t** | ***p*** |
| **Among all participants** | -0.052 | 0.025 | -0.153 | -2.079 | **0.039** | -0.157 | 0.062 | -0.289 | -2.544 | **0.013** | -0.005 | 0.002 | -0.267 | -2.403 | **0.019** |
| **Among Aβ- NCs** | -0.111 | 0.13 | -0.098 | -0.856 | 0.395 | -0.152 | 0.171 | -0.144 | -0.886 | 0.381 | 0.001 | 0.006 | 0.026 | 0.165 | 0.870 |
| **Among Aβ+ NCs** | -0.036 | 0.097 | -0.05 | -0.369 | 0.714 | -0.308 | 0.137 | -0.399 | -2.244 | **0.034** | -0.008 | 0.005 | -0.314 | -1.607 | **0.121** |
| **Among aMCI plus ADD groups** | -0.013 | 0.028 | -0.067 | -0.481 | 0.633 | -0.015 | 0.081 | -0.048 | -0.185 | 0.858 | 0.000 | 0.004 | 0.013 | 0.042 | 0.967 |
|  | **Baseline HP volume** | | | | | **Annualized change of HP/TIV ratio** | | | | | **Annualized change of HP volume (%)** | | | | |
|  | **β** | **SE** | **Standard β** | **t** | ***p*** | **β** | **SE** | **Standard β** | **t** | ***p*** | **β** | **SE** | **Standard β** | **t** | ***p*** |
| **Among all participants** | -0.054 | 0.047 | -0.086 | -1.143 | 0.254 | -0.057 | 0.055 | -0.121 | -1.029 | 0.307 | -0.001 | 0.001 | -0.084 | -0.739 | 0.462 |
| **Among Aβ- NCs** | -0.105 | 0.205 | -0.059 | -0.512 | 0.61 | -0.137 | 0.150 | -0.149 | -0.917 | 0.365 | 0.004 | 0.003 | 0.172 | 1.072 | 0.291 |
| **Among Aβ+ NCs** | 0.008 | 0.149 | 0.007 | 0.051 | 0.959 | -0.093 | 0.114 | -0.162 | -0.819 | 0.421 | 0.001 | 0.003 | 0.060 | 0.316 | 0.755 |
| **Among aMCI plus ADD groups** | 0.015 | 0.071 | 0.029 | 0.212 | 0.833 | 0.019 | 0.096 | 0.059 | 0.203 | 0.843 | 0.001 | 0.003 | 0.115 | 0.351 | 0.734 |
|  | **Baseline PCC volume** | | | | | **Annualized change of PCC/TIV ratio** | | | | | **Annualized change of PCC volume (%)** | | | | |
|  | **β** | **SE** | **Standard β** | **t** | ***p*** | **β** | **SE** | **Standard β** | **t** | ***p*** | **β** | **SE** | **Standard β** | **t** | ***p*** |
| **Among all participants** | 0.061 | 0.034 | 0.138 | 1.795 | 0.074 | -0.072 | 0.072 | -0.119 | -1.003 | 0.319 | 0.000 | 0.001 | -0.012 | -0.097 | 0.923 |
| **Among Aβ- NCs** | 0.062 | 0.19 | 0.039 | 0.324 | 0.747 | -0.511 | 0.205 | -0.372 | -2.496 | **0.017** | -0.002 | 0.003 | -0.133 | -0.864 | 0.393 |
| **Among Aβ+ NCs** | 0.008 | 0.147 | 0.007 | 0.057 | 0.954 | 0.003 | 0.167 | 0.004 | 0.017 | 0.986 | 0.003 | 0.003 | 0.237 | 1.160 | 0.257 |
| **Among aMCI plus ADD groups** | 0.052 | 0.033 | 0.224 | 1.567 | 0.124 | 0.093 | 0.051 | 0.416 | 1.814 | 0.103 | 0.003 | 0.002 | 0.511 | 1.704 | 0.123 |
|  | **Baseline Pre volume** | | | | | **Annualized change of Pre/TIV ratio** | | | | | **Annualized change of Pre volume (%)** | | | | |
|  | **β** | **SE** | **Standard β** | **t** | ***p*** | **β** | **SE** | **Standard β** | **t** | ***p*** | **β** | **SE** | **Standard β** | **t** | ***p*** |
| **Among all participants** | 0.146 | 0.081 | 0.138 | 1.804 | 0.073 | -0.045 | 0.202 | -0.026 | -0.222 | 0.825 | 0.001 | 0.001 | 0.074 | 0.633 | 0.529 |
| **Among Aβ- NCs** | 0.185 | 0.448 | 0.05 | 0.413 | 0.681 | -0.774 | 0.529 | -0.229 | -1.463 | 0.152 | 0.002 | 0.003 | 0.089 | 0.572 | 0.571 |
| **Among Aβ+ NCs** | 0.032 | 0.353 | 0.012 | 0.091 | 0.928 | 0.107 | 0.526 | 0.043 | 0.204 | 0.840 | 0.003 | 0.004 | 0.183 | 0.869 | 0.393 |
| **Among aMCI plus ADD groups** | 0.13 | 0.083 | 0.223 | 1.572 | 0.123 | 0.264 | 0.212 | 0.260 | 1.241 | 0.246 | 0.003 | 0.002 | 0.535 | 1.934 | 0.085 |
|  | **Baseline GM volume** | | | | | **Annualized change of GM/TIV ratio** | | | | | **Annualized change of GM volume (%)** | | | | |
|  | **β** | **SE** | **Standard β** | **t** | ***p*** | **β** | **SE** | **Standard β** | **t** | ***p*** | **β** | **SE** | **Standard β** | **t** | ***p*** |
| **Among all participants** | 0.117 | 0.419 | 0.013 | 0.28 | 0.78 | -0.044 | 0.041 | -0.123 | -1.069 | 0.288 | 0.000 | 0.001 | 0.011 | 0.100 | 0.921 |
| **Among Aβ- NCs** | -0.706 | 1.732 | -0.023 | -0.408 | 0.685 | -0.147 | 0.120 | -0.196 | -1.230 | 0.226 | 0.003 | 0.003 | 0.177 | 1.196 | 0.239 |
| **Among Aβ+ NCs** | 1.67 | 1.139 | 0.082 | 1.466 | 0.149 | -0.135 | 0.090 | -0.311 | -1.504 | 0.146 | 0.000 | 0.004 | -0.017 | -0.083 | 0.935 |
| **Among aMCI plus ADD groups** | 1.071 | 0.54 | 0.19 | 1.984 | **0.053** | 0.018 | 0.071 | 0.071 | 0.261 | 0.800 | 0.001 | 0.001 | 0.316 | 0.938 | 0.373 |

Analyses were performed for different groups. For the first analysis, nEV Aβ_42_, age, sex, TIV, and *APOE* ε4 status were used as predictors of baseline brain regional volume. For the second analysis, nEV Aβ_42_, age, sex, and *APOE* ε4 status were used as predictors of annualized changes in brain regional volume (expressed as the ratio to corresponding TIV; therefore, the TIV did not need to be additionally included). For the third analysis, nEV Aβ_42_, age, sex, and *APOE* ε4 status were used as predictors of annualized changes in brain regional volume (expressed as the percentage of changes; therefore, the TIV did not need to be additionally included). Regression results for age, sex, TIV, and *APOE* ε4 status are not listed here. There were 192 subjects who had baseline sMRI assessments, including 75 Aβ- NCs, 62 Aβ+ NCs, 27 aMCI, and 28 ADD individuals, and 88 subjects who had follow-up sMRI assessments, including 43 Aβ- NCs, 31 Aβ+ NCs, 11 aMCI, and three ADD individuals. The average follow-up time was 13.26 ± 4.7 months.

Abbreviations: Aβ, β-amyloid; NCs, cognitively normal controls; aMCI, amnestic mild cognitive impairment; ADD, Alzheimer’s disease dementia; nEV, neuronal-derived extracellular vesicle; APOE, apolipoprotein E; Ent, entorhinal cortex; HP, hippocampus; PCC, posterior cingulate; Pre, precuneus; GM, grey matter; TIV, total intracranial volume; SE, standard error; sMRI, structural magnetic resonance imaging.


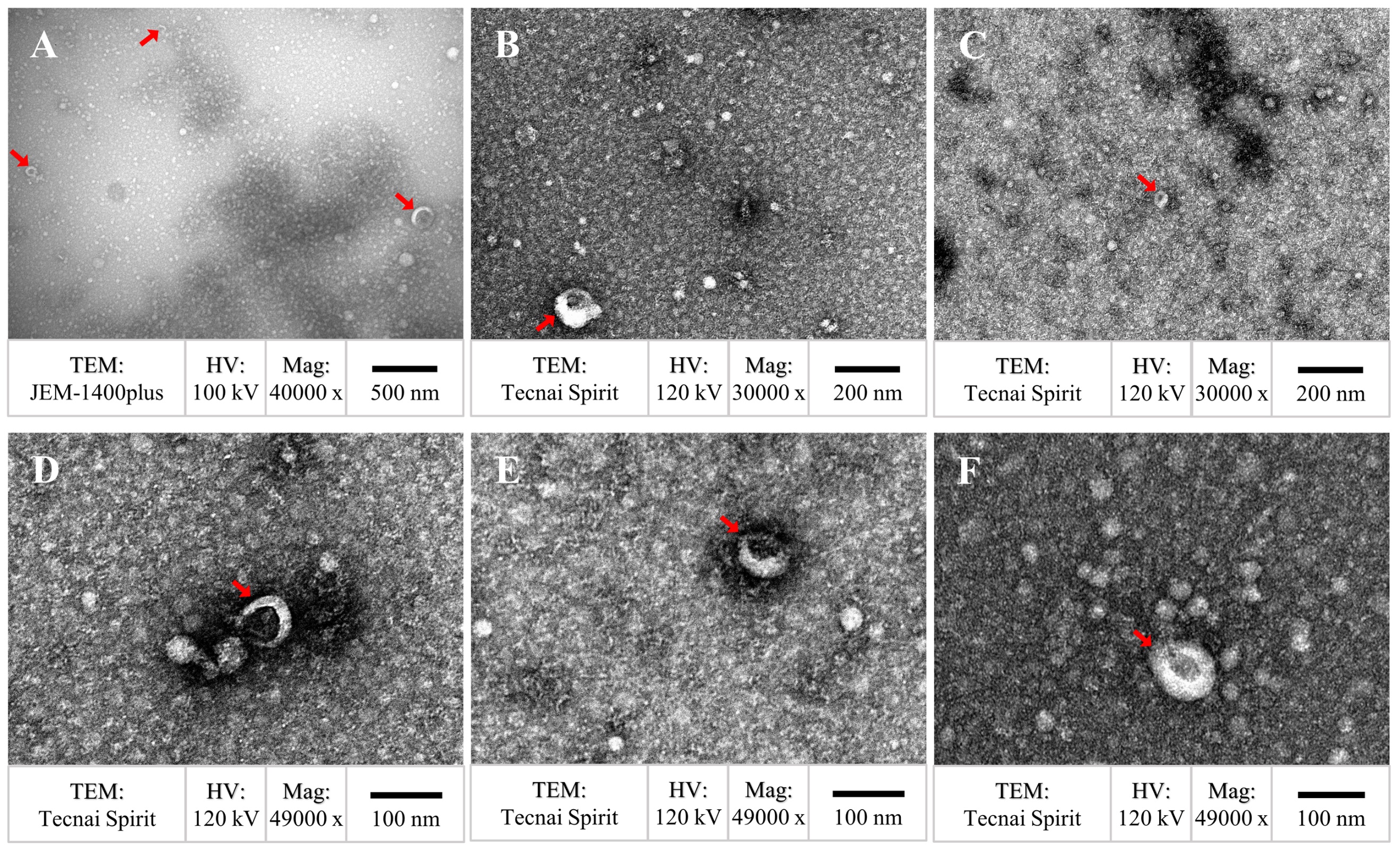


**Supplementary Figure 1. Typical TEM images of nEV.**

Characteristic images of nEV from six subjects in different magnifications (red arrow). The analysis was performed in two different TEMs from Capital medical university (**A**) and Tsinghua University (**B-F**), respectively.

Abbreviations: TEM, transmission electron microscope; HV, high voltage; Mag, magnification; nEV, neuronal-derived extracellular vesicle.


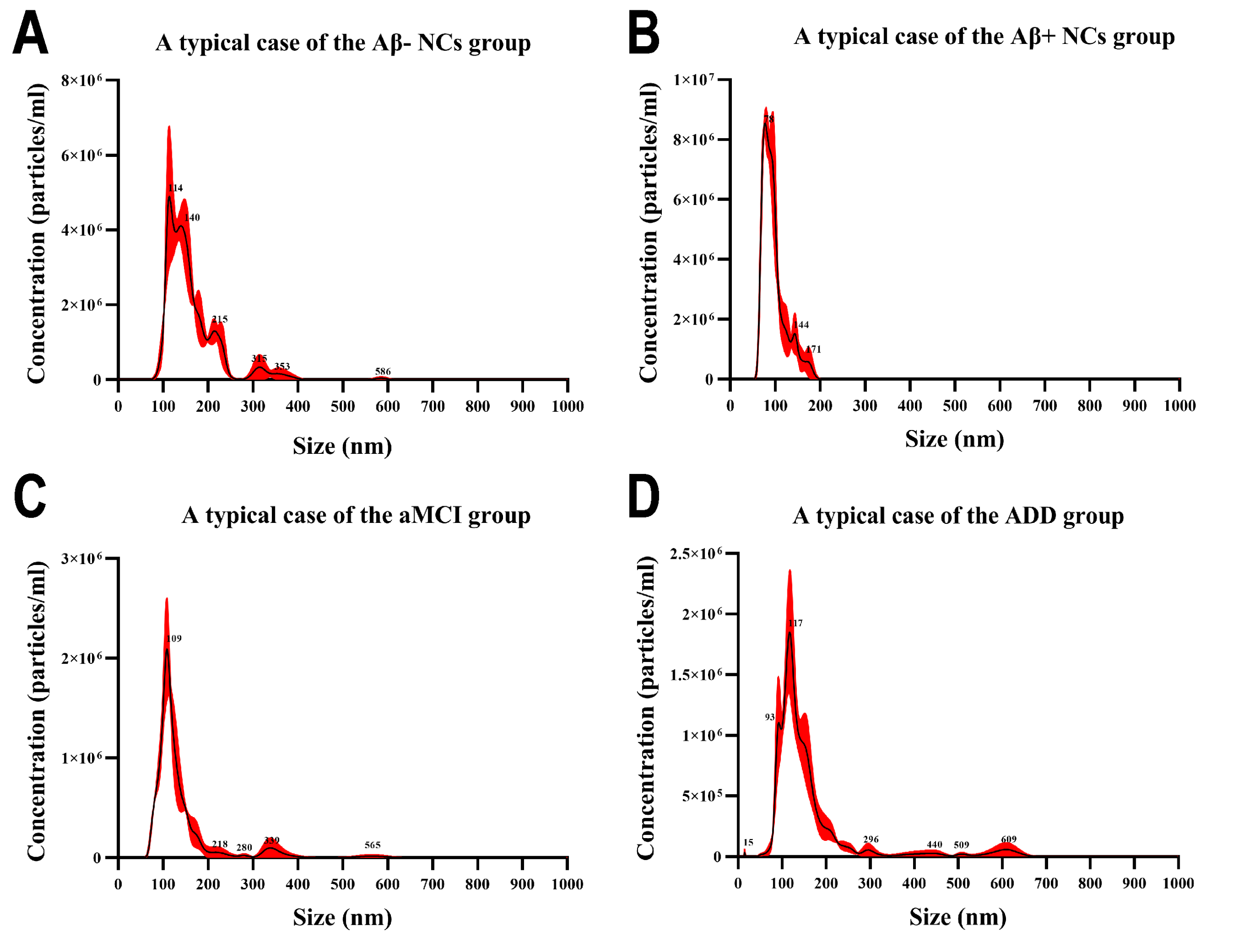


**Supplementary Figure 2. Typical NTA results.**

The graphs display particle concentration (particles/ml) against size (diameter in nm) of four subjects with different diagnoses.

Abbreviations: NTA, nanoparticle tracking analysis; Aβ, β-amyloid; NCs, cognitively normal controls; aMCI, amnestic mild cognitive impairment; ADD, Alzheimer’s disease dementia.


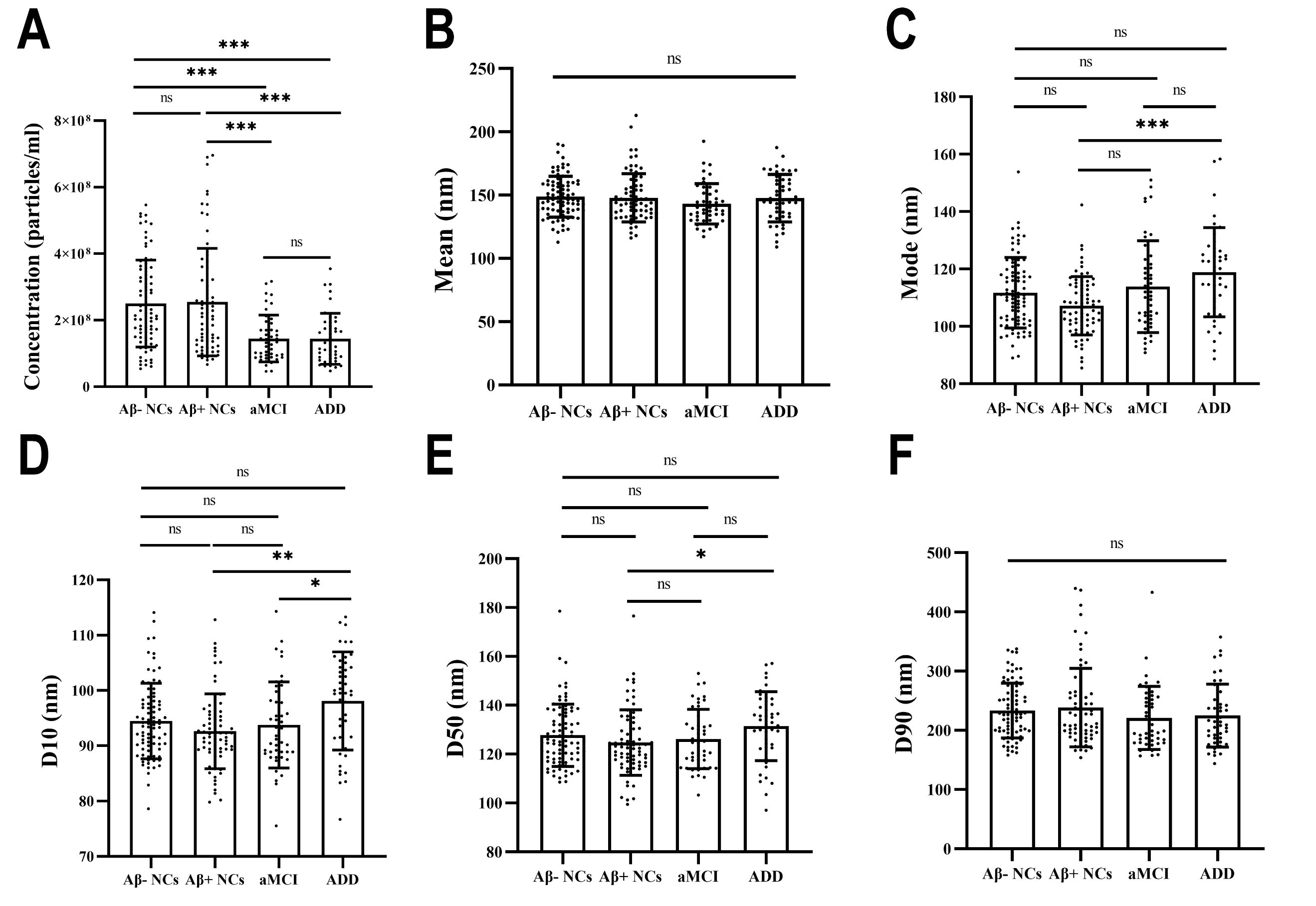


**Supplementary Figure 3. NTA results of enrolled subjects.**

The NTA analyses were performed by the same researcher on the same instrument of the same parameters. The statistical analysis was conducted by Kruskal‒Wallis H test, followed by multiple post hoc comparisons (adjusted *p* value). Comparisons: * *p* < 0.05; ** *p* < 0.01; *** *p* < 0.001; ns, > 0.05. Notably, these results were obtained directly after dilution.

Abbreviations: NTA, nanoparticle tracking analysis; Aβ, β-amyloid; NCs, cognitively normal controls; aMCI, amnestic mild cognitive impairment; ADD, Alzheimer’s disease dementia.


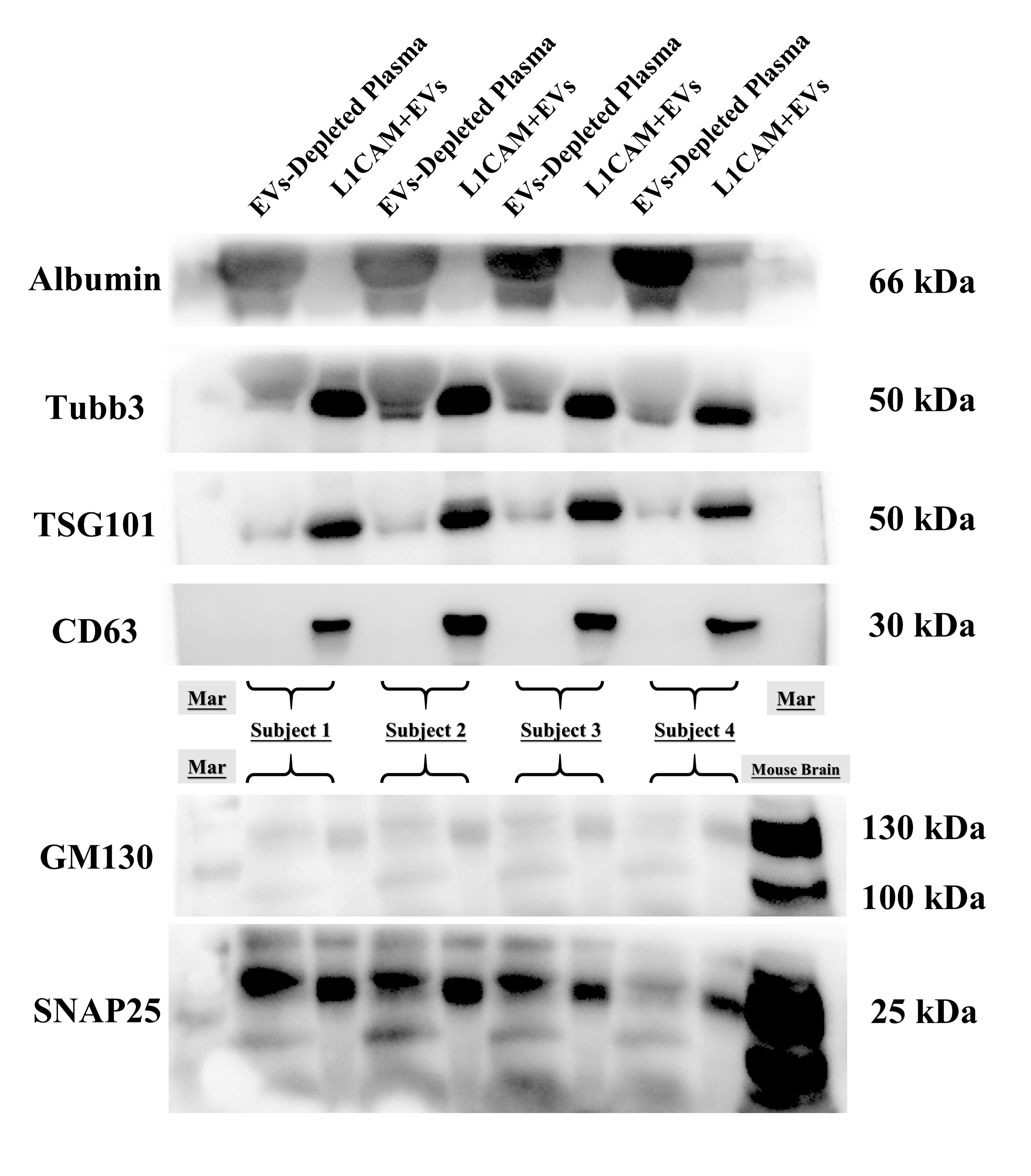


**Supplementary Figure 4. Western blot characterization of nEVs.**

Western blots of neuronal-enriched (L1CAM+) plasma EVs compared to EV-depleted plasma (supernatant obtained after Exoquick®) from four subjects. First, compared with the supernatant, nEVs had obviously higher CD63 and TSG101 levels, demonstrating the nature of EVs. Second, compared to the supernatant, nEVs had lower albumin levels. In addition, GM130 was not clearly observed in nEVs, suggesting purity. Third, TUBB3 and SNAP25, two classic neuronal markers, were clearly observed in nEVs, suggesting a true neuronal origin.

Abbreviations: EVs, extracellular vesicles; nEVs, neuronal-derived EVs; Mar, marker; L1CAM, L1 cell adhesion molecular (CD171).


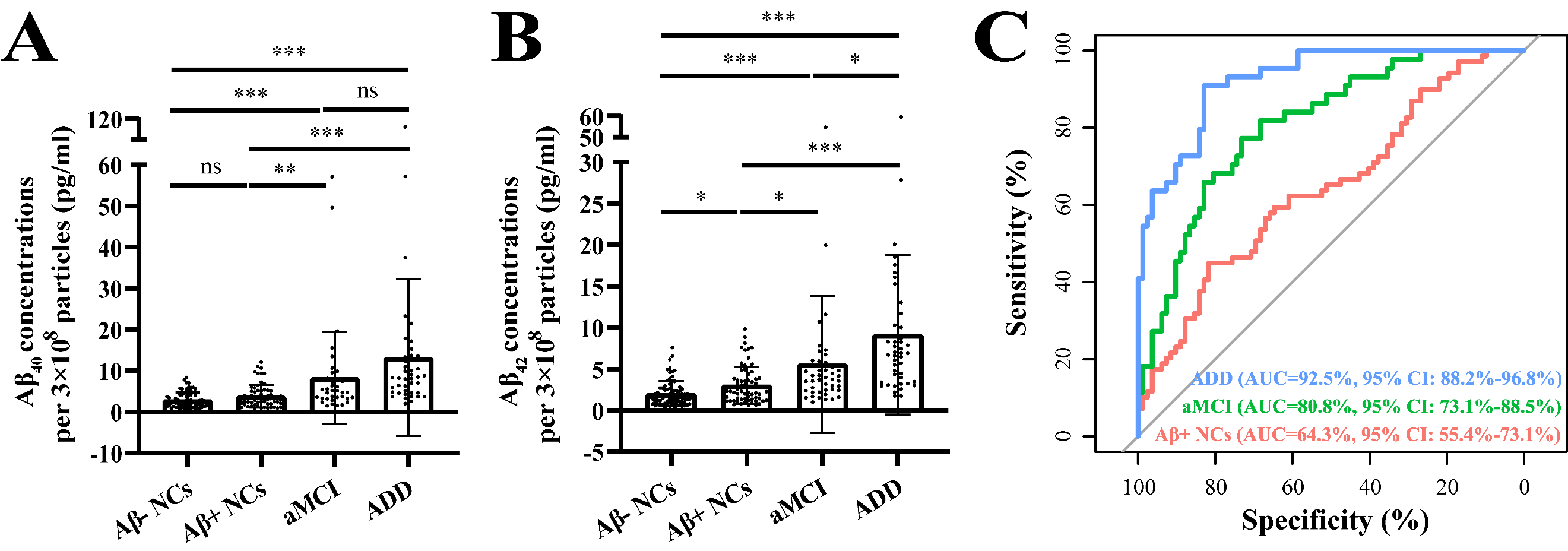


**Supplementary Figure 5. nEV Aβ concentrations in different diagnostic groups and the ROC curves.**

The analysis was performed after correcting the particle numbers to 3 × 10^8^ for nEV Aβ_40_ (**A**) and nEV Aβ_42_ (**B**). All the outliers were included in the analysis. Statistical analysis was conducted using the Kruskal‒Wallis H test, followed by multiple post hoc comparisons (adjusted *p* value). Comparisons among groups: * *p* < 0.05; ** *p* < 0.01; *** *p* < 0.001; ns, > 0.05. Furthermore, ROC curves were used to distinguish Aβ- NCs from Aβ+ NCs (red), aMCI (green), and ADD (blue) using nEV Aβ_42_ (**C**). All outliers were excluded. Outliers were defined as less than Q1 - 2.5×IQR or greater than Q3 + 2.5×IQR.

Abbreviations: Aβ, β-amyloid; NCs, cognitively normal controls; aMCI, amnestic mild cognitive impairment; ADD, Alzheimer’s disease dementia; nEV, neuronal-derived extracellular vesicle; AUC, area under the curve; ROC, receiver operating characteristic; CI, confidence interval; IQR, inter-quartile range; Q1, lower quartile; Q3, upper quartile.


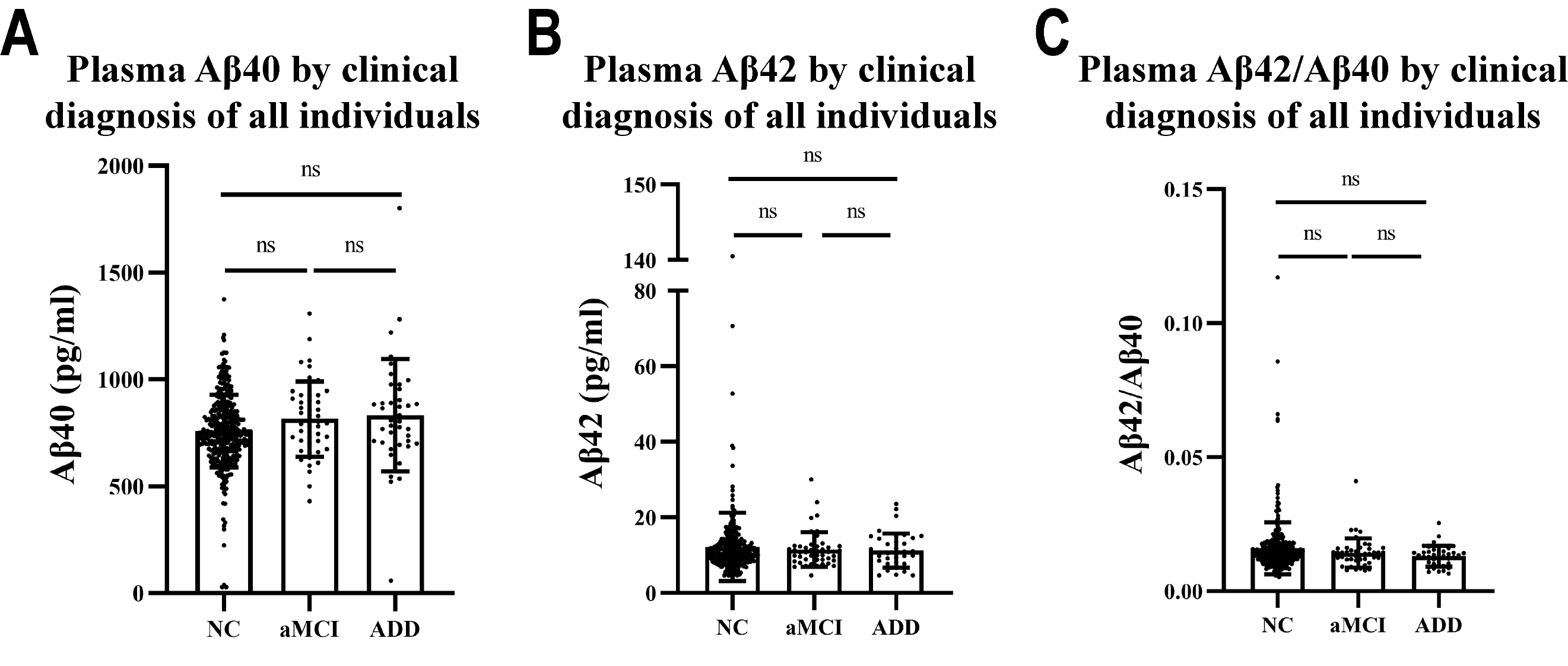


**Supplementary Figure 6. Plasma Aβ concentrations in different diagnostic groups.**

The corresponding clinical data for these patients are shown in **Supplementary Table 4**. Statistical analysis was conducted using Kruskal‒Wallis H test, followed by multiple post hoc comparisons (adjusted *p* value). Diagnoses were made based on clinical information, and there were no differences in plasma Aβ_40_ (**A**), Aβ_42_ (**B**), and Aβ_42_/Aβ_40_ ratio (**C**) among the different groups. ns, > 0.05.

Abbreviations: Aβ, β-amyloid; NC, cognitively normal control; aMCI, amnestic mild cognitive impairment; ADD, Alzheimer’s disease dementia.


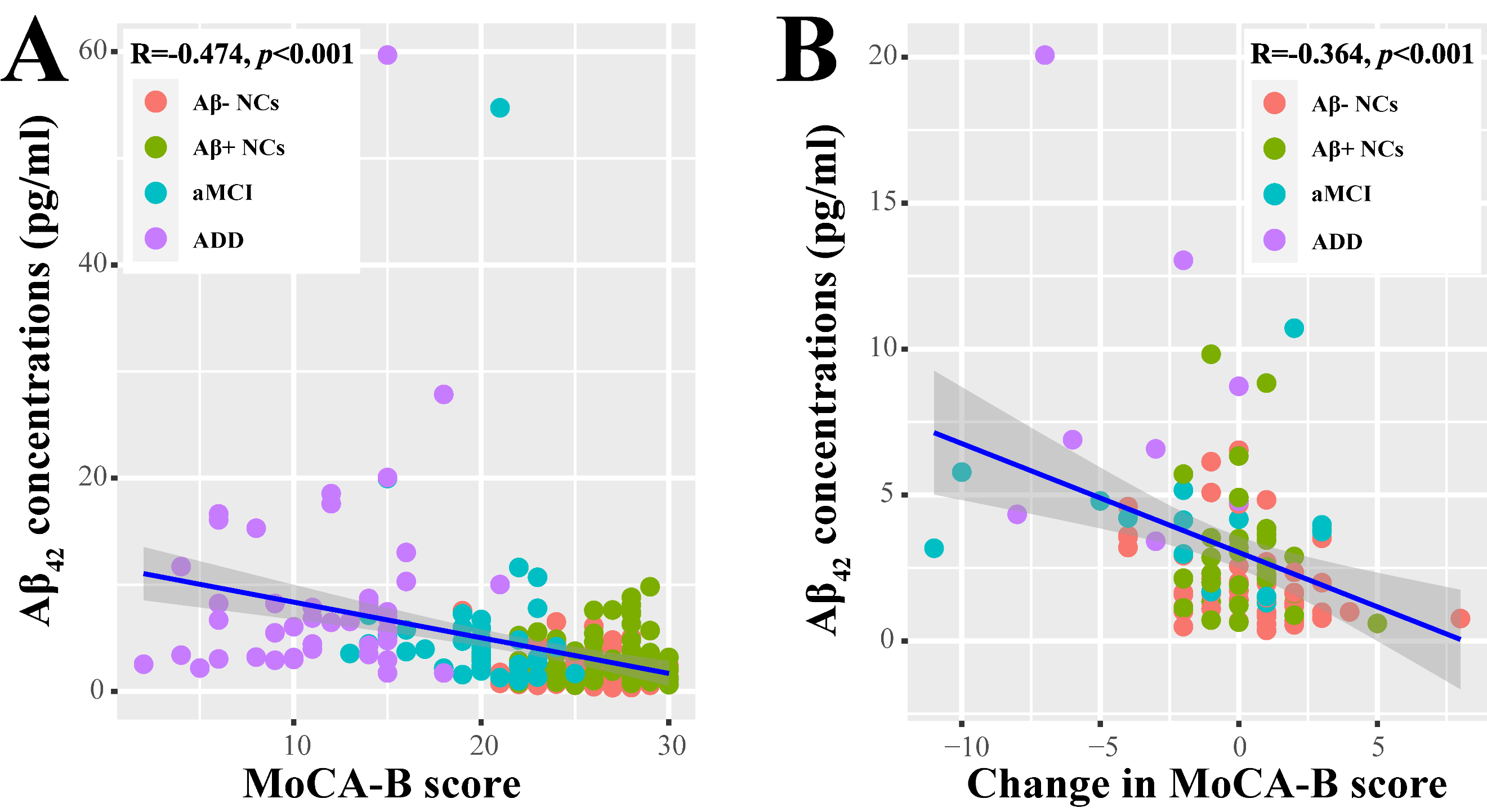


**Supplementary Figure 7. Association between nEV Aβ_42_ concentrations and MoCA-B scale.**

(**A**) Correlation analysis was performed between the nEV Aβ_42_ concentrations and baseline MoCA-B scores. (**B**) Plots showing the subset of individuals who had follow-up cognitive assessments (104 subjects, including 49 Aβ- NCs, 33 Aβ+ NCs, 14 aMCI, and eight ADD individuals, with an average follow-up time of 14.58 ± 6.37 months). Correlation analysis was performed between the nEV Aβ_42_ concentrations and longitudinal changes in the MoCA-B scores. The correlation coefficients and *p* values are listed.

Abbreviations: Aβ, β-amyloid; NCs, cognitively normal controls; aMCI, amnestic mild cognitive impairment; ADD, Alzheimer’s disease dementia; nEV, neuronal-derived extracellular vesicle; MoCA-B, Montreal cognitive assessment-basic version.


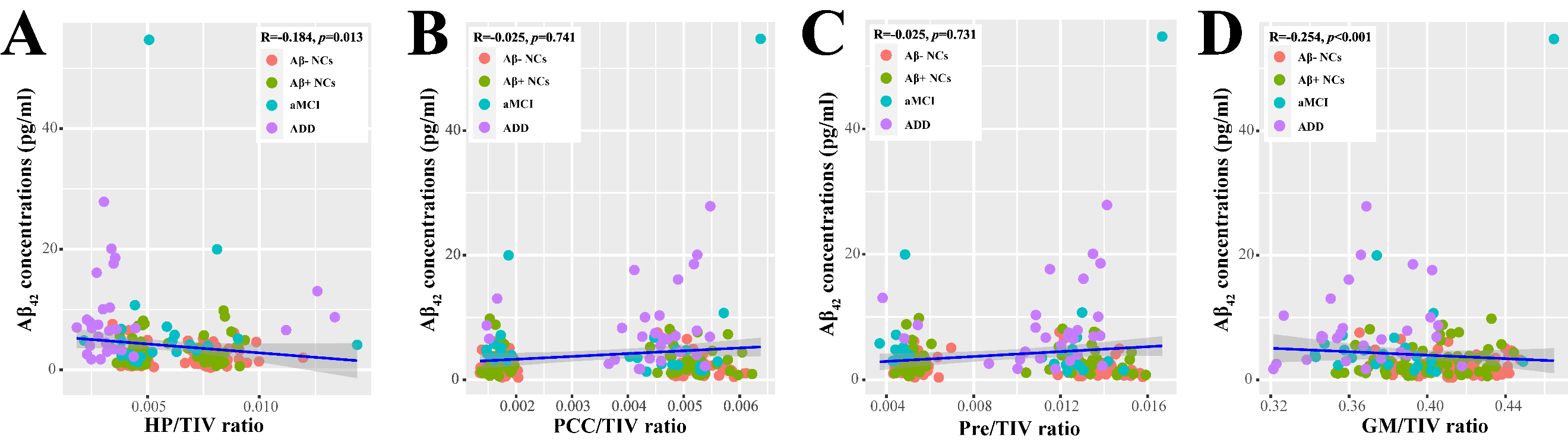


**Supplementary Figure 8. Association between nEV Aβ_42_ concentrations and baseline brain regional volume.**

Correlation analysis was performed between nEV Aβ_42_ concentrations and baseline brain regional volumes, including those of the HP (**A**), PCC (**B**), Pre (**C**), and GM (**D**). The volumes are expressed as the ratio of the corresponding TIVs. The correlation coefficients and *p* values are also listed. The analyses were performed in 192 subjects who had baseline sMRI assessments, including 75 Aβ- NCs, 62 Aβ+ NCs, 27 aMCI, and 28 ADD individuals.

Abbreviations: Aβ, β-amyloid; NCs, cognitively normal controls; aMCI, amnestic mild cognitive impairment; ADD, Alzheimer’s disease dementia; nEV, neuronal-derived extracellular vesicle; HP, hippocampus; PCC, posterior cingulate; Pre, precuneus; GM, grey matter; TIV, total intracranial volume; sMRI, structural magnetic resonance imaging.


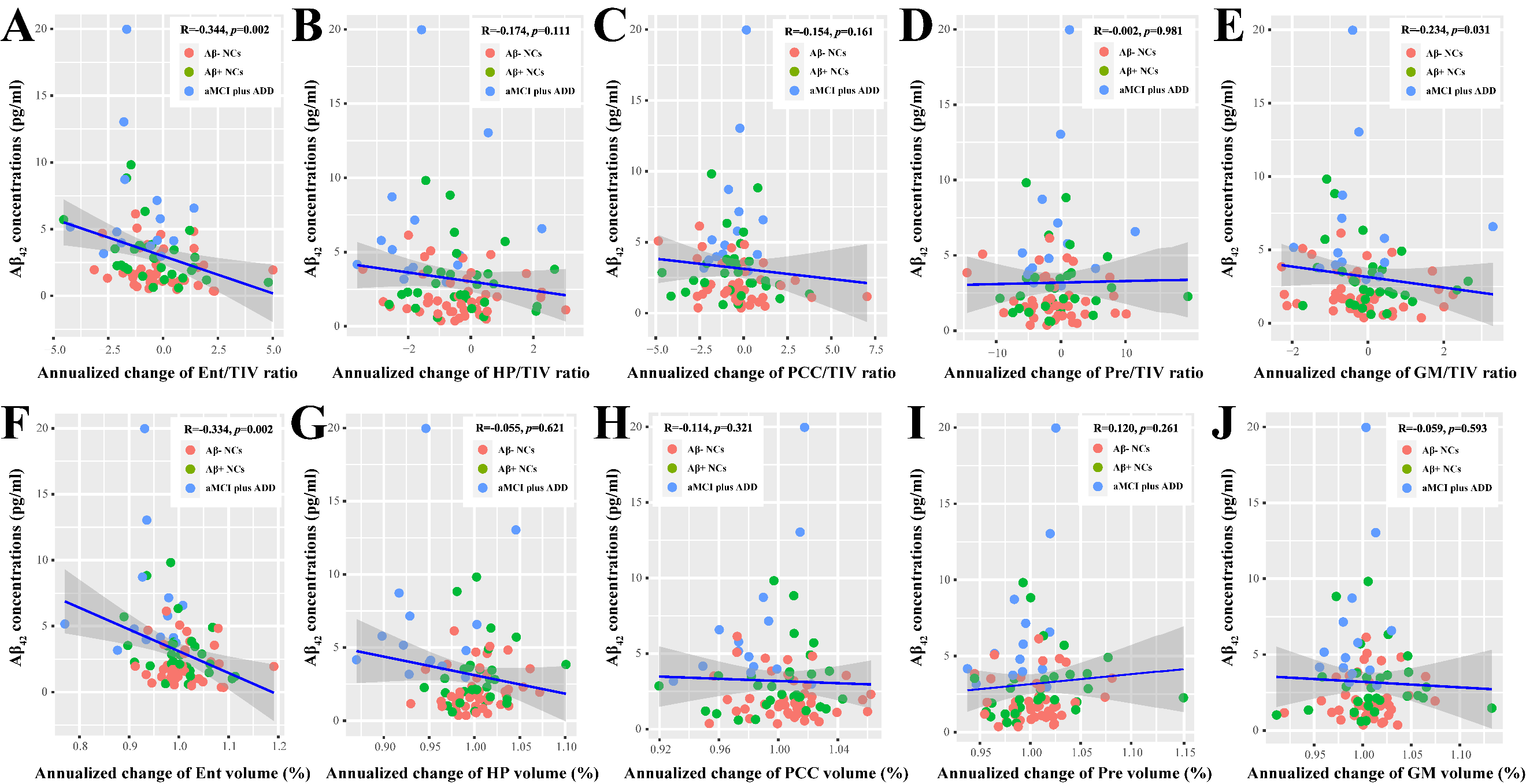


**Supplementary Figure 9. Association between nEV Aβ_42_ concentrations and longitudinal changes in brain regional volume.**

Correlation analysis was performed between baseline nEV Aβ_42_ concentrations and longitudinal changes in brain regional volumes, including those of the Ent (**A, F**), HP (**B, G**), PCC (**C, H**), Pre (**D, I**), and GM (**E, J**). The longitudinal changes in brain regional volumes were annualized and expressed as the ratio to corresponding TIVs (**A‒E**; multiplied by 10^4^ of Ent, HP, PCC, and Pre; multiplied by 10^2^ of GM) or expressed as the percentage of changes (**F‒J**). The analyses were performed in 88 subjects who had follow-up sMRI assessments, including 43 Aβ- NCs, 31 Aβ+ NCs, 11 aMCI, and three ADD individuals. The average follow-up time was 13.26 ± 4.7 months. The correlation coefficients and *p* values are also listed.

Abbreviations: Aβ, β-amyloid; NCs, cognitively normal controls; aMCI, amnestic mild cognitive impairment; ADD, Alzheimer’s disease dementia; nEV, neuronal-derived extracellular vesicle; Ent, entorhinal cortex; HP, hippocampus; PCC, posterior cingulate; Pre, precuneus; GM, grey matter; TIV, total intracranial volume; sMRI, structural magnetic resonance imaging.

**References**

1. Li TR, Dong QY, Jiang XY, Kang GX, Li X, Xie YY, et al. Exploring brain glucose metabolic patterns in cognitively normal adults at risk of Alzheimer's disease: A cross-validation study with Chinese and ADNI cohorts. Neuroimage Clin. 2021;33:102900.

2. Li TR, Wu Y, Jiang JJ, Lin H, Han CL, Jiang JH, et al. Radiomics Analysis of Magnetic Resonance Imaging Facilitates the Identification of Preclinical Alzheimer's Disease: An Exploratory Study. Front Cell Dev Biol. 2020;8:605734.

3. Du W, Ding C, Jiang J, Han Y. Women Exhibit Lower Global Left Frontal Cortex Connectivity Among Cognitively Unimpaired Elderly Individuals: A Pilot Study from SILCODE. J Alzheimers Dis. 2021;83(2):653-63.

4. Dong QY, Li TR, Jiang XY, Wang XN, Han Y, Jiang JH. Glucose metabolism in the right middle temporal gyrus could be a potential biomarker for subjective cognitive decline: a study of a Han population. Alzheimers Res Ther. 2021;13(1):74.

5. Li X, Wang X, Su L, Hu X, Han Y. Sino Longitudinal Study on Cognitive Decline (SILCODE): protocol for a Chinese longitudinal observational study to develop risk prediction models of conversion to mild cognitive impairment in individuals with subjective cognitive decline. BMJ Open. 2019;9(7):e028188.

6. Gonzalez-Escamilla G, Lange C, Teipel S, Buchert R, Grothe MJ, Alzheimer's Disease Neuroimaging Initiative. PETPVE12: an SPM toolbox for Partial Volume Effects correction in brain PET - Application to amyloid imaging with AV45-PET. Neuroimage. 2017;147:669-77.

7. Long JM, Holtzman DM. Alzheimer Disease: An Update on Pathobiology and Treatment Strategies. Cell. 2019;179(2):312-39.

8. Farokhian F, Beheshti I, Sone D, Matsuda H. Comparing CAT12 and VBM8 for Detecting Brain Morphological Abnormalities in Temporal Lobe Epilepsy. Front Neurol. 2017;8:428.

9. Tavares V, Prata D, Ferreira HA. Comparing SPM12 and CAT12 segmentation pipelines: a brain tissue volume-based age and Alzheimer's disease study. J Neurosci Methods. 2019;334:108565.

10. Fellhauer I, Zöllner FG, Schröder J, Degen C, Kong L, Essig M, et al. Comparison of automated brain segmentation using a brain phantom and patients with early Alzheimer's dementia or mild cognitive impairment. Psychiatry Res. 2015;233(3):299-305.

11. Chaves H, Dorr F, Costa ME, Serra MM, Slezak DF, Farez MF, et al. Brain volumes quantification from MRI in healthy controls: Assessing correlation, agreement and robustness of a convolutional neural network-based software against FreeSurfer, CAT12 and FSL. J Neuroradiol. 2021;48(3):147-56.

12. Shearn A, Aday S, Ben-Aicha S, Carnell-Morris P, Siupa A, Angelini GD, et al. Analysis of Neat Biofluids Obtained During Cardiac Surgery Using Nanoparticle Tracking Analysis: Methodological Considerations. Front Cell Dev Biol. 2020;8:367.

13. Pulliam L, Sun B, Mustapic M, Chawla S, Kapogiannis D. Plasma neuronal exosomes serve as biomarkers of cognitive impairment in HIV infection and Alzheimer's disease. J Neurovirol. 2019;25(5):702-9.

14. Kapogiannis D, Mustapic M, Shardell MD, Berkowitz ST, Diehl TC, Spangler RD, et al. Association of Extracellular Vesicle Biomarkers With Alzheimer Disease in the Baltimore Longitudinal Study of Aging. JAMA Neurol. 2019;76(11):1340-51.

15. Fiandaca MS, Kapogiannis D, Mapstone M, Boxer A, Eitan E, Schwartz JB, et al. Identification of preclinical Alzheimer's disease by a profile of pathogenic proteins in neurally derived blood exosomes: A case-control study. Alzheimers Dement. 2015;11(6):600-7.e1.

16. Goetzl EJ, Mustapic M, Kapogiannis D, Eitan E, Lobach IV, Goetzl L, et al. Cargo proteins of plasma astrocyte-derived exosomes in Alzheimer's disease. FASEB J. 2016;30(11):3853-9.

17. Winston CN, Goetzl EJ, Akers JC, Carter BS, Rockenstein EM, Galasko D, et al. Prediction of conversion from mild cognitive impairment to dementia with neuronally derived blood exosome protein profile. Alzheimers Dement (Amst). 2016;3:63-72.

18. Jia L, Qiu Q, Zhang H, Chu L, Du Y, Zhang J, et al. Concordance between the assessment of Aβ42, T-tau, and P-T181-tau in peripheral blood neuronal-derived exosomes and cerebrospinal fluid. Alzheimers Dement. 2019;15(8):1071-80.

19. Shi M, Kovac A, Korff A, Cook TJ, Ginghina C, Bullock KM, et al. CNS tau efflux via exosomes is likely increased in Parkinson's disease but not in Alzheimer's disease. Alzheimers Dement. 2016;12(11):1125-31.

20. Guix FX, Corbett GT, Cha DJ, Mustapic M, Liu W, Mengel D, et al. Detection of Aggregation-Competent Tau in Neuron-Derived Extracellular Vesicles. Int J Mol Sci. 2018;19(3).

21. Nam E, Lee YB, Moon C, Chang KA. Serum Tau Proteins as Potential Biomarkers for the Assessment of Alzheimer's Disease Progression. Int J Mol Sci. 2020;21(14).

22. Winston CN, Goetzl EJ, Baker LD, Vitiello MV, Rissman RA. Growth Hormone-Releasing Hormone Modulation of Neuronal Exosome Biomarkers in Mild Cognitive Impairment. J Alzheimers Dis. 2018;66(3):971-81.

23. Zhao A, Li Y, Yan Y, Qiu Y, Li B, Xu W, et al. Increased prediction value of biomarker combinations for the conversion of mild cognitive impairment to Alzheimer's dementia. Transl Neurodegener. 2020;9(1):30.

24. Eren E, Hunt J, Shardell M, Chawla S, Tran J, Gu J, et al. Extracellular vesicle biomarkers of Alzheimer's disease associated with sub-clinical cognitive decline in late middle age. Alzheimers Dement. 2020;16(9):1293-304.

25. Delgado-Peraza F, Nogueras-Ortiz CJ, Volpert O, Liu D, Goetzl EJ, Mattson MP, et al. Neuronal and Astrocytic Extracellular Vesicle Biomarkers in Blood Reflect Brain Pathology in Mouse Models of Alzheimer's Disease. Cells. 2021;10(5).
